# Genomic evaluation of hybridization in historic and modern North American Bison (*Bison bison*)

**DOI:** 10.1038/s41598-022-09828-z

**Published:** 2022-04-16

**Authors:** Sam Stroupe, David Forgacs, Andrew Harris, James N. Derr, Brian W. Davis

**Affiliations:** 1 Department of Veterinary Pathobiology, Texas A&M University College of Veterinary Medicine and Biomedical Science, College Station, TX 77843 USA; 2 Interdisciplinary Graduate Program of Genetics, Texas A&M University, College Station, TX 77843 USA; 3 Department of Veterinary Integrative Biosciences, Texas A&M University College of Veterinary Medicine and Biomedical Science, College Station, TX 77843 USA; 4 Department of Small Animal Clinical Sciences, Texas A&M University College of Veterinary Medicine and Biomedical Science, College Station, TX 77843 USA; 5 Present Address: Center for Vaccines and Immunology, University of Georgia, Athens, GA 30605 USA

**Keywords:** Population genetics, Agricultural genetics, Animal breeding, Genetic hybridization

## Abstract

During the late nineteenth century North American bison underwent a significant population bottleneck resulting in a reduction in population size of over 99% and a species-level near-extinction event. Factors responsible for this destruction included indiscriminate killing, loss of access to suitable habitat, and diseases. At the nadir of this population crash, very few wild plains bison survived and were restricted to Yellowstone National Park, USA and a small number of wild wood bison remained in Wood Buffalo National Park, Canada. However, most surviving bison in the late 1800’s were maintained by cattle ranchers in private herds where hybridization between bison with various breeds of domestic cattle was often encouraged. Over the last 20 years, the legacy of this introgression has been identified using mitochondrial DNA and limited nuclear microsatellite analyses. However, no genome-wide assessment has been performed, and some herds were believed to be free of introgression based on current genetic testing strategies. Herein, we report detailed analyses using whole genome sequencing from nineteen modern and six historical bison, chosen to represent the major lineages of bison, to identify and quantitate signatures of nuclear introgression in their recent (within 200 years) history. Both low and high coverage genomes provided evidence for recent introgression, including animals from Yellowstone, Wind Cave, and Elk Island National Parks which were previously thought to be free from hybridization with domestic cattle. We employed multiple approaches, including one developed for this work, to identify putative cattle haplotypes in each bison genome. These regions vary greatly in size and frequency by sample and herd, though we detected domestic cattle introgression in all bison genomes tested. Since our sampling strategy spanned across the diversity of modern bison populations, these finding are best explained by multiple historical hybridization events between these two species with significant genetic recombination over the last 200 years. Our results demonstrate that whole genome sequencing approaches are required to accurately quantitate cattle introgression in bison.

*“There is reason to fear that unless the United States Government takes the matter in hand and makes a special effort to prevent it, the pure-blood bison will be lost irretrievably through mixture with domestic breeds and through in-and-in breeding.” (Hornaday 1889)*

## Introduction

North American bison are a modern conservation success story and among the most unique species due to their multiple roles in society. They are considered livestock and propagated for meat and fiber production, wildlife on public lands, revered as a religious and cultural symbol among some Native American peoples, and as the National Mammal of the United States of America. Bison diverged from the cattle lineage about 2.5–3.7 Mya, according to nuclear and mitochondrial DNA evidence^1^. Bison ancestors arrived in North America as early as 135,000–195,000 years ago via Beringia then subsequently expanded to cover much of the continent, giving rise to modern North American bison, *Bison bison*^2^. Today, this species includes two recognized sub-species, plains bison, *B. b. bison* and wood bison, *B. b. athabascae*.

At the height of their recorded population history, bison numbered in the millions within their range from Canada to northern Mexico^3^. However, in the late 1800s, they experienced a devastating population bottleneck due to indiscriminate killing, loss of access to suitable habitat, and death by epidemic caused by exposure to imported and native infectious diseases^4–6^. The salvation of the species is owed to a few ranchers from Texas to Canada who established bison herds with captured wild calves during the nadir of the population crash. Without the foresight of these ranchers, bison may be extinct today. However, these ranchers were intrinsically cattlemen, which consequently led to the organized efforts to hybridize bison with various domestic cattle breeds^7–10^. Some of these ranchers sought to breed a more profitable animal by deliberately hybridizing bison with their cattle, while others allowed hybridization to happen by raising the two species together. Additionally, earlier attempts to domesticate and hybridize bison with cattle are known to have occurred during the mid 1700s^11,12^. These efforts prompted the noted conservationist, William T. Hornaday, to warn of the potential consequence that hybridization can have on the integrity of bison as a species^11,12^. Nevertheless, hybridization experiments between these two species continued into the 1900s.

**Figure 1:**
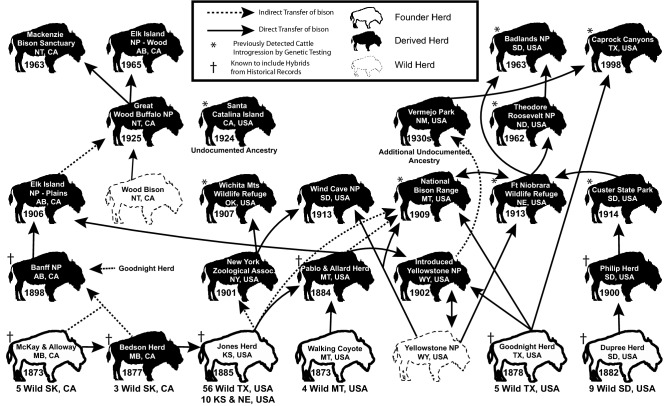
This graph represents the survival and repopulation of bison by tracking the major founding lineages from wild captured bison to modern populations.The wild herds depicted represent the only populations of bison that survived the population bottleneck in the wild. The founder herds were privately owned bison that were established with bison captured from the wild. The wild and founder herds, seven in total, are the only herds that were in existence during the nadir of the population crash and represent the seven lineages of bison. The derived herds are those that were established with bison from one or more of the seven lineages. The solid arrow (direct transfer) represents direct movement of bison from one population to another, while the dashed line (indirect transfer) represents movement of bison with one or more intermediate populations between the populations depicted. This graph does not include all bison movement but is instead meant to show the contribution and influence that the founding herds have on modern populations based on documented evidence of transferring bison. This figure also identifies populations that are known to have domestic cattle introgression whether through historical documentation or modern genetic testing and the years when each population was established. The Banff National Park population is not the same as the reintroduced animals that currently reside in the park^16,17–19^.

Beginning in the 1870s, bison went through a complex and interconnected history of human mediated movement to propagate the species (Fig. 1). Following the apex of the population bottleneck, most surviving bison were under private ownership, limiting the number of bison available to establish new public and private herds^11,13^. On selecting bison for these new populations, it was noted that after four or five generations of backcrossing, hybrids are visually indistinguishable from the non-hybridized parental species^11^. Bison derived from these privately-owned herds were also used to augment the only two remaining wild populations, Yellowstone National Park (*B. b. bison*) and in the Northwest Territories of Canada, now Wood Buffalo National Park (*B. b. athabascae*)^13–15^. While much of the bison movement between populations has been thoroughly recorded, especially within public herds, there are still herds whose origins or additions are from undocumented sources^16,17–19^.

**Figure 2:**
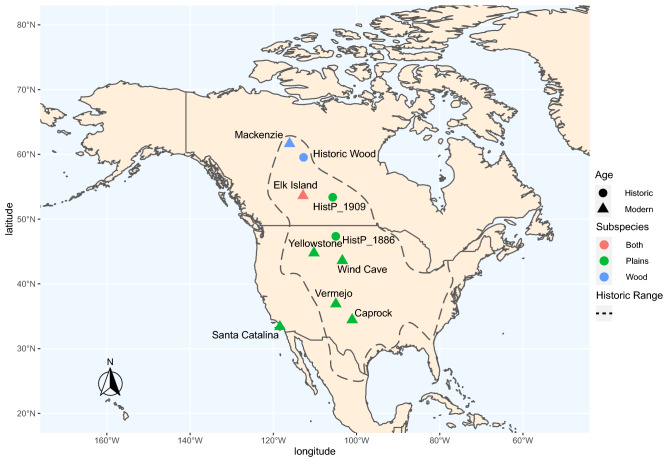
Map of sampled bison populations. Color-coded by subspecies (Plains or Wood). Shapes are used to differentiate historic (before 1940) and modern (after 1990) samples. The dotted line estimates the historic range of bison in North America^12^. Image generated in R v4 using bisonmap.R script at github.com/agaricx/bisonmap/.

More than a century after these hybridization events occurred, domestic cattle mitochondrial haplotypes were discovered in modern bison populations^18,19^. Since that time, numerous private and public bison herds have been examined extensively to document evidence of hybridization. These approaches were limited to identification of matrilineal cattle ancestry using mitochondrial DNA sequencing, as well as a limited number of nuclear microsatellite markers^20–22^. Using these methods, many herds appeared to be free from cattle introgression, however none were genome-wide approaches. To examine the genomic differences between ancestral and modern bison more thoroughly and identify specific individuals and herds with residual signatures of introgression, we sequenced the genomes of nineteen modern and six historical bison across six distinct North American herds (Fig. 2) and compared them to a panel of over 1,842 cattle from a wide variety of breeds. We identified an excess of shared derived alleles between cattle and bison^23^, tested for patterns of regional variation consistent with recent hybridization^24^, and identified specific variant alleles with a high likelihood of cattle origin using IntrogressionID, an algorithm developed for this work.

## Results

Approximately 26.2 million biallelic Single Nucleotide Polymorphisms (SNPs) were identified across 25 bison genomes (Table 1). 93.5 million SNPs were found when adding one individual from four cattle breeds (Angus, Charolais, Hereford, and Holstein) and water buffalo (*Bubalus bubalus*). Multidimensional scaling (MDS) using a subset of 4.3 million SNPs and fastSTRUCTURE admixture analyses using a subset of 8.8 million SNPs confirmed bison and cattle as distinct populations, with definitive division between multiple bison herds (Extended Data Figs. 1a,b and 2). Of the historical and modern bison examined, the number of likely distinct populations was five (k = 5) (Extended Data Fig. 1b). Historical plains bison, all modern samples from Yellowstone National Park and Vermejo Park Ranch, and one individual each from Santa Catalina Island and Wind Cave National Park comprised the largest group, with historic wood bison, Elk Island National Park wood bison, and Mackenzie Bison Sanctuary wood bison forming another group. MDS confirmed this bifurcation between the northern and southern latitudes for both historical and modern bison herds (Extended Data Fig. 1a).

**Table 1:** Locations and sources of material used for whole genome sequencing and comparison. Date of sampling and subspecies are shown. All samples were obtained opportunistically. Bone and hide samples were donated from their curated collection. Hair and blood were obtained from annual round-ups from the ranchers responsible for the management of the herd. No animals were accessed solely to sample for this study.

Sample	Population	Source	Collection Date	Subspecies
Caprock_01	Caprock Canyons State Park (TX, USA)	Texas Parks & Wildlife	2018	Plains
Caprock_02	Caprock Canyons State Park (TX, USA)	Texas Parks & Wildlife	2018	Plains
ElkIslandP_01	Elk Island National Park—Plains (AB, Canada)	Parks Canada	2018	Plains
ElkIslandP_02	Elk Island National Park—Plains (AB, Canada)	Parks Canada	2018	Plains
ElkIslandW_01	Elk Island National Park—Wood (AB, Canada)	Parks Canada	2018	Wood
ElkIslandW_02	Elk Island National Park—Wood (AB, Canada)	Parks Canada	2018	Wood
HistP_1886	Historical Plains Bison (MT, USA)	Smithsonian Institution	1886	Plains
HistP_1909	Historical Plains Bison (SK, Canada)	Canadian Museum of Nature	1909	Plains
HistW_1892	Historical Wood Bison (AB/NT, Canada)	Canadian Museum of Nature	1892	Wood
HistW_1921	Historical Wood Bison (AB/NT, Canada)	Parks Canada	1921	Wood
HistW_1937	Historical Wood Bison (AB/NT, Canada)	Smithsonian Institution	1937	Wood
Mackenzie_01	Mackenzie Bison Sanctuary (NT, Canada)	Parks Canada	1999	Wood
Mackenzie_02	Mackenzie Bison Sanctuary (NT, Canada)	Parks Canada	1999	Wood
SantaCataIsl_01	Santa Catalina Island (CA, USA)	Santa Catalina Island Conservancy	2009	Plains
SantaCataIsl_02	Santa Catalina Island (CA, USA)	Santa Catalina Island Conservancy	2009	Plains
Vermejo_01	Vermejo Park Ranch (NM, USA)	Turner Enterprises, Inc	2000	Plains
Vermejo_02	Vermejo Park Ranch (NM, USA)	Turner Enterprises, Inc	2001	Plains
WindCave_01	Wind Cave National Park (SD, USA)	US National Parks Service	2018	Plains
WindCave_02	Wind Cave National Park (SD, USA)	US National Parks Service	2018	Plains
Yellowstone_01	Yellowstone National Park (WY/MT, USA)	US National Parks Service	2011	Plains
Yellowstone_02	Yellowstone National Park (WY/MT, USA)	Turner Enterprises, Inc	2011	Plains
Yellowstone_03	Yellowstone National Park (WY/MT, USA)	US National Parks Service	2000	Plains
Yellowstone_04	Yellowstone National Park (WY/MT, USA)	US National Parks Service	2000	Plains
Yellowstone_05	Yellowstone National Park (WY/MT, USA)	US National Parks Service	2009	Plains
Yellowstone_1925	Yellowstone National Park (WY/MT, USA)	Yellowstone Heritage and Research Center	1925	Plains
Angus_01	NCBI	SRR1425124	–	Cattle
Charolais_01	NCBI	SRR1355258	–	Cattle
Hereford_01	NCBI	SRR2226524	–	Cattle
Holstein_01	NCBI	SRR1365147	–	Cattle
*Bubalus bubalis*	NCBI	SRR7284794	–	Water Buffalo

**Figure 3:**
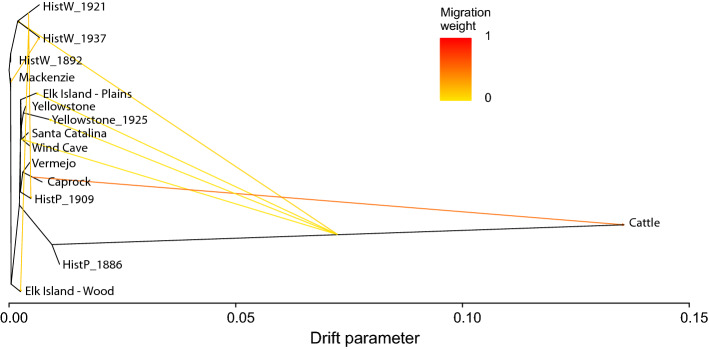
TreeMix estimation of phylogenetic network and relationships among bison populations and domestic cattle with eight migration events. All modern bison were assigned to their respective populations while historic samples were included as individuals. A representative of each of the four cattle breeds (Angus, Charolais, Hereford, and Holstein) were assigned to the cattle population. Of the migration edges five of the eight are between the cattle lineage and bison populations. The migration with the highest weight is shown between cattle and Caprock while migrations of smaller weights can be seen between Cattle and historical wood bison, Elk Island-Plains, Yellowstone and a group consisting of Wind Cave and Santa Catalina. Three additional migration events can be seen between historical wood bison and modern wood bison populations as well as between historical plains and wood bison. These migration events shown agree with the historic documentation of introgression events and movements of bison.

In general, the overall biallelic heterozygosity within historical and modern individuals varied widely (Supplementary Table 1). Though low-depth individuals (~ 10 × and below) suffer from heterozygote detection deficiency (Extended Data Fig. 3), those with higher depth do not show a correlation with sample age (*p* < 0.01 between modern and historical) (Supplementary Table 1). Directional gene flow between cattle and all contemporary populations of bison was evident when modeling migration events as applied to the background maximum likelihood phylogeny (Fig. 3). In Fig. 3, five of the eight migration events are between the cattle lineage and bison populations. The migration with the highest weight is shown between Cattle and Caprock while migrations of smaller weights can be seen between Cattle and historical wood bison, Elk Island- Plains, Yellowstone and a group consisting of Wind Cave and Santa Catalina. Three additional migration events can be seen between historical wood bison and modern wood bison populations as well as between historical plains and wood samples. These migration events shown in Fig. 3, agree with the documentation of introgression and movements of bison.

**Figure 4:**
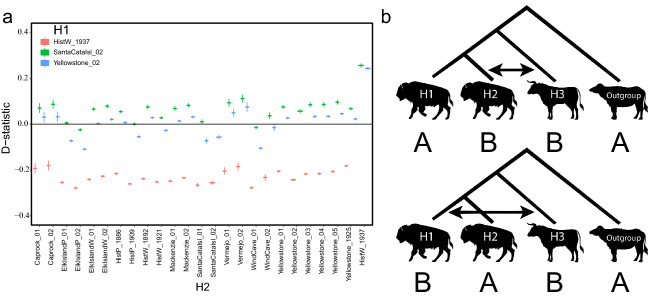
(**a**) D-statistics from individual level all-to-all comparisons of ancestral and derived allele frequencies. Negative D-statistic indicates that H1 (bison) is closer to H3 (Angus cattle) then H2 (bison). (**b**) Phylogenetic tree to show potential gene flow between *B. taurus* (H3) and *B. bison* (H1) or *B. bison* (H2).

Comparison of ancestral and derived allele frequencies by calculation of individual-level Patterson’s D, or D-statistic, using all variation demonstrated that all bison sequenced possess evidence for introgression using water buffalo as an outgroup. Though the degree of significance varied considerably by sample, the all-to-all individual level comparison allowed the determination of the difference between D calculated when each bison was used as H1 or H2 (Supplementary Table 2). The emerging pattern suggested that the most highly introgressed individual was a historical wood bison from 1937, with consistent detection of introgression in all other samples (Fig. 4).

However, Patterson’s *D* statistic was designed to detect evidence of hybridization but does not specifically quantify levels of introgression. Therefore, identification of genomic regions from introgressed origin was performed using two approaches: HybridCheck and IntrogressionID (IID). The HybridCheck algorithm evaluates samples for signals of introgression by identifying changes in sequence similarity using a sliding-window^24^. Each bison was compared to the ARS1.2 *Bos taurus* reference genome and a bison from each modern population, in triplicates, across all autosomes. Introgression blocks were determined by regions in which the sequence similarity was greater between *Bos taurus* than other bison. Average sequence similarities are recorded in Table S3. This approach identified cattle introgressed chromosomal regions in all 25 bison, in each independent comparison (Table S4). To remove potential comparison biases, only regions that overlapped in at least five of the eight comparisons per sample were kept as regions of potential introgression. In addition, 29 regions totaling 3.7 Mb were identified as introgressed in every comparison, the largest of these stretching over 336 Kb on chromosome 21 (Supplementary Table 5). These overlapping regions are potentially due to repetitive sequence causing low mapping resolution, primarily in telomeric regions, and were not considered evidence for introgression (Supplementary Table 6). Two regions of note are immediately downstream of the bovine major histocompatibility complex (MHC) class II region, DRB3 (BoLA-DRB3), a region that has been associated with susceptibility to several infectious diseases^25,26^.

This method identified the lowest levels of autosomal introgression in Yellowstone_02 with 0.24% cattle and the highest levels in HistW_1937 with 2.45%. Caprock Canyons State Park samples have on average longer maximum introgressed blocks than any other modern population, stretching over 11 Mb, in addition to multiple much smaller individual blocks broken up by generations of recombination (Supplementary Table 6). Sequencing depth appears to have little influence on the positive identification of hybridization signal, as similar percentage introgression was identified for both samples from Caprock Canyons State Park, which were 36.6 × and 4.2 × average autosomal coverage. While sequencing depth could have a slight effect on the detection of introgression, even in other populations where samples have large variation in sequencing depth, the presence of introgression was identified in both samples (Supplementary Tables 1, 6).

The second, IntrogressionID, is a novel method developed for this work. In this analysis, introgression is identified by private introgressed variation present in a heterozygous state in a single bison. In this model, a SNP must be fixed for the reference allele in all cattle and fixed for the alternate allele in all other bison. Therefore, the bison in question will be heterozygous with one copy of the cattle allele and the other copy being the bison allele. This extremely aggressive model was designed to detect introgression with high confidence by taking advantage of fixed alleles in each species. Using this method is an effective way to eliminate signals that could be due to incomplete lineage sorting or ancestral polymorphism. This model was ran using a panel of 1,842 cattle whole genomes (from www.1000bullgenomes.com) and our 25 bison.

**Figure 5:**
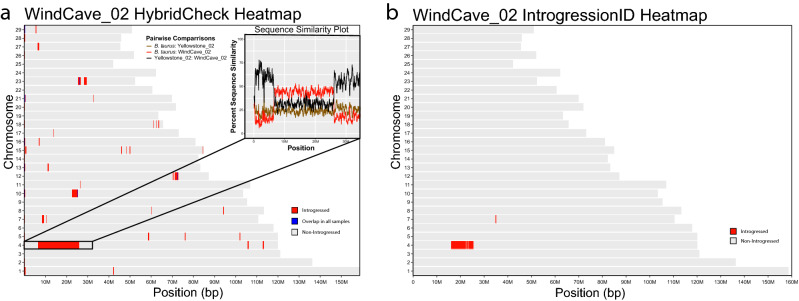
Whole genome heatmap of detected introgressed blocks by HybridCheck (**a**) and IntrogressionID (**b**) for individual WindCave_02. Chromosomes are listed numerically along the y-axis and their lengths are represented by the length of the respective heatmap box along the x-axis. Across each chromosome, the grey coloration indicates regions of no signal for introgression, the red coloration indicates signal of introgression and the blue coloration in (**a**) indicates signals of introgression that overlap across all sample comparisons for HybridCheck. The zoomed in box shows the HybridCheck output data plotted in a line graph with percent sequence similarity across the y-axis and base pair position across the x-axis.

Populations previously thought to be free of hybridization showed considerable signatures of introgression, such as Wind Cave National Park, using both methods. Figure 5 depicts the distribution of introgressed regions across the genome of WindCave_02 using HybridCheck (Fig. 5a) and IntrogressionID (Fig. 5b). A large introgressed region detected by both methodologies can be seen along a section of chromosome 4. This region was the largest detected among all samples. Regions of introgression for each sample as detected by HybridCheck and IntrogressionID can be seen in Extended Data Figs. 4 and 5.

## Discussion

North American bison are iconic among native species due to their multfaceted status as a production species, a wildlife species, a spiritual reference species and as the National Mammal of the United States. Moreover, their reputation is further enhanced by the well-documented catastrophic decimation this species endured in the later part of the nineteenth century and their spectacular recovery to over 500,000 bison today scattered across hundreds of public, private, and non-governmental agency herds. Without question, North American bison as a species are resilient and tenacious in their ability to survive and eventually thrive despite significant population-level obstacles. This genomic evaluation of historical levels of introgression between bison and domestic cattle was designed to investigate and help preserve the genetic integrity of this species.

For the last 20 years, domestic cattle introgression into bison populations was investigated using mitochondrial DNA (mtDNA) sequencing and a set of 14 nuclear microsatellite markers that were shown to have non-overlapping alleles in the two species^19,22^. Both molecular approaches have considerable limits in their ability to detect introgressed regions. mtDNA is limited to detecting only unbroken maternal lineages from a domestic cow and the limited number of validated nuclear microsatellites restrict sampling to a small percentage of the genome^19,22^. Nevertheless, numerous population level studies using these methods uncovered evidence of domestic cattle introgression in most bison populations. With the resolution provided by high-throughput whole genome sequencing, we were able to interrogate each complete bison genome at the single nucleotide level. Following robust and detailed approaches, we found that every bison herd examined, including Yellowstone, Wind Cave, and Elk Island (plains and wood bison) National Parks that have been previously believed to be free from cattle introgression, all have detectable levels of hybrid ancestry with cattle (Supplementary Table 6, Extended Data Figs. 4 and 5). These results were confirmed by examination of individual-level D-statistics that revealed significant cattle ancestry in all samples tested (Fig. 5, Supplementary Table 2). However, since ancestral hybridization is known to have occurred in multiple bovid species, this could have some effect on the significance of the D-statistics^1^. Nevertheless, introgression detected with HybridCheck and IntrogressionID show recent hybridization events as evident by large genomic regions with signals of introgression. In addition, detection of introgression blocks using these methods of allele frequency and private variation approaches identified ubiquitous presence of cattle genomic regions in each individual bison sampled. Genomic regions varied widely in size, from 400 bp to over 11 Mb, and frequency, from 35 to over 300 detected regions, between samples and herds, but no sample was completely free of apparent cattle genetic signal (Supplementary Table 6). Bison are not a panmictic population but are made up of many isolated herds with only human-mediated migration events. Therefore, the variation of introgression seen is in concordance with each herd having their own unique history. This detectable cattle introgression may increase variation in heterozygous regions in bison, however with only a small portion of the genome containing cattle alleles is unlikely to have any significant effect. Further analysis would need to be done to test for evidence of selection using a larger sample size.

As depicted in Fig. 1, the movement of bison across North America has been interconnected and mediated by humans since the late 1800s. Our sampling strategy was designed to include bison that represent all historical founder bison lineages that were established in the late 1800s. Well-documented and detailed record keeping has given us a clear history of how bison survived the bottleneck and once again re-populated North America^11,13^. Each of the original founding populations have a unique history, and unfortunately, they all have a history of bison-cattle hybridization or the translocation and incorporation of cross-bred bison into their herds.

Historical evidence of the origin of all cattle introgression is difficult to pinpoint, but four of the five founder herd owners (Dupree (US), Goodnight (US), Jones (US) and McKay & Alloway (CA) herds) were actively involved in hybridization experimentation (Fig. 1). The only exception was Walking Coyote (US), whose founding herd was not known to have included hybrid bison. However, he sold his herd to Pablo & Allard who subsequently purchased hybrid bison from Jones and incorporated these animals into that herd (Fig. 1). Historical records alone suggest no major extant bison lineage or population is likely to be completely isolated from exposure to cattle introgression. This is underscored in the First Annual Report of the American Bison Society, that stated there were hybrids in Banff National Park, Goodnight, Allard-derived, Philip, and Jones herds. In fact, approximately 2% of all captive bison in North America were known hybrids according this 1908 population count^11^.

The evidence of cattle introgression in all historical bison samples, especially those that predate the major hybridization events, was surprising. Traces of recent hybridization in samples such as HistW_1937 and Yellowstone_1925 can be explained by the introduction of bison derived from private owners, who practiced hybridization, into the population before these samples were collected. However, samples such as HistP_1886 and HistW_1902 do not have a simple explanation for signals of introgression. These bison were members of what were considered some of the last remaining wild bison populations in the US and Canada at the time. Since these samples were collected before the well documented hybridization and subsequent distribution of bison as previously mentioned, this leaves two possible explanations for the detected introgression in these historical samples: 1. There were multiple earlier hybridization events that went unrecorded, and the hybrids were released/escaped into the wild or 2. Some domestic cattle brought to North America by European settlers escaped and feralized, joining wild bison herds, leading to subsequent hybridization.

There is documentation that supports the likelihood of each scenario. Though brief, it comes from credible sources. Reports of small numbers of domesticated bison in captivity along the Eastern coast of the US from Pennsylvania and south to the Carolinas can be traced back to the 1700s. Included in these reports is the earliest documented account of bison-cattle hybridization, which occurred before 1750^27^. Audubon also documented a case of hybridizing domestic cattle with wild captured bison prior to 1843^28^. Both accounts are much earlier than previously thought and predate all bison samples included in this study. Additionally, Hornaday affirmed that it was not unheard of for escaped domestic cattle to permanently join wild bison herds^12^. While these sporadic and experiential hybridization events between bison and cattle occurred in small herds, the genetic contributions the resulting animals made to modern bison populations is unclear. The detection of introgression in these historical samples, however, suggests that additional unrecorded hybridization events occurred between the arrival of domestic cattle with European settlers and the well-documented recent history of bison / cattle hybridization experiments of the late 1800s.

Though we developed IID specifically for this system, it has broad application to any set of species with adequate population-level data. With growing awareness of hybridization in both captivity and the wild, IID provides a framework in which fundamental questions about the presence of introgression can be addressed. For example, IID can identify signatures of recent introgression between domestic cat and wild cats used in the pet trade, as well as wolf-dogs and wolf-coyote hybrids [unpublished data]. Since it relies on population-scale allele frequency data and is aggressive in eliminating signals of incomplete lineage sorting, there are considerable applications for natural populations such as those of polar/grizzly bears and multiple cetaceans affected by climate change^29,30^. The use of IID is only limited by one's ability to collect population-level data from their species of interest.

## Conclusions

This genome-wide investigation comparing 25 bison, selected to represent the major lineages following the population bottleneck in the nineteenth century, to domestic cattle, uncovered evidence for multiple hybridization events between these two species over the last 200 years. Our methods included three independent approaches to document and evaluate domestic cattle introgression into modern bison genomes including one in which bison were compared to a panel of 1,842 domestic cattle. Detection of introgression was not hampered by low sequencing depth and all bison genomes evaluated were found to possess multiple and unique genomic regions of cattle origin. While the possibility exists that there are still some bison individuals that are free of cattle introgression, it is highly unlikely any of the large public, private, tribal, or non-governmental organization herds have escaped this fate.

Examples of hybridization between closely related mammalian species are common and have been documented for numerous species^30–33^. However, examples of introgressive hybridization based on whole genome sequence analyses between two distinct mammalian genera are rare^34^. European bison (*Bison bonasus*) and North American bison were both originally placed in the genus *Bos* by Linnaeus but subsequently separated into the genus *Bison* as reviewed by McDonald 1981^35^. In fact, a recent genomic evaluation of the phylogenetic relationships among members of the genus *Bos*^36^, found extensive hybridization among various species in this genus and clear taxonomic support for both European bison and North American bison within the genus *Bos* and not separated in their own genus *Bison*. Moreover, they documented introgression among most members of the genus *Bos* and proposed this as a new source of adaptive variation facilitating domestication and in response to environmental changes.

Documentation of species-wide introgression of cattle genomic regions across all major bison lineages will impact long-term conservation efforts in numerous ways. To date, genetic conservation in bison has centered on preserving genetic diversity through long-term management policies while limiting exposure to hybrid bison defined by a limited number of species defining microsatellite loci and mitochondrial DNA sequences. The realization that relatively low levels of cattle introgression is pervasive across the species should enhance opportunities for broader and more inclusive species-wide conservation priorities for public, private, tribal and NGO managed bison populations.

Multiple recent and ongoing investigations to fully annotate the bison genome^37,38^ will provide the necessary bison genomic reference information to investigate specific regions and genes and identify signatures of genomic selection. Due to the size and uniquely distributed genomic regions of introgression, methods utilizing reduced representation such as RADseq would likely not detect all regions. Additionally, because our results show that introgression can be detected across both high and low coverage sequences, a low pass sequencing approach would be the most cost-effective, accurate, and reproducible method moving forward. An increase in the number of available bison genome sequences will allow for the study of population variation and structure, leading to an understanding of the pervasiveness of cattle genomic contributions to modern bison.

## Methods

### Sampling

Our sampling strategy was designed to include bison that represent all the historical founder bison lineages that were established in the late 1800s. Hair, blood, tissue, or bone samples from historical and modern bison from across North America were collected from museum holdings or from bison populations (Table 1). All samples were obtained opportunistically. Bone and hide samples were donated from their curated collection. Hair and blood were obtained from annual round-ups from the ranchers responsible for the management of the herd. No animals were accessed solely to sample for this study. All permissions were obtained from the museums and protected areas to collect the samples.

### Sequencing and variant calling

DNA was extracted at the DNA Technologies Core Lab at Texas A&M University based on previously published protocols^39,40^. The six historical bison samples were handled and stored separately from the modern samples and DNA was extracted in a UV killing isolation station with dedicated equipment and reagents as described by Curry and Derr^39^. Most of the genomic libraries were prepared and sequenced by Delta Genomics (Edmonton, Alberta, Canada) using the methods described by Yang et al.^41^. Additional genomic libraries (Mackenzie Bison Sanctuary, Vermejo Park Ranch, and Yellowstone_1925) were prepared by Texas A&M AgriLife Genomics and Bioinformatics Service (College Station, TX) and sequenced using the Illumina NovaSeq 6000 platform. The average genome coverages and mapping quality are listed in Supplementary Table 1.

In addition to bison, one high sequencing depth cattle (*Bos taurus*) genome from four breeds (Angus, Charolais, Hereford, and Holstein) and one water buffalo (*Bubalus bubalis*) were selected for analysis from the NCBI database (Table 1). The cattle breeds were chosen strategically to cover breeds that were known to be used in bison-cattle hybridization experiments^7,9^. Using default parameters for all algorithms, FASTQ data was aligned to the *Bos taurus* genome build ARS-UCD1.2 using SpeedSeq^42^, sorted and indexed by SAMtools 1.3.1^43^, and PCR duplicates marked with PicardTools^44^. In cases where samples were run on multiple lanes, they were merged using SAMtools^43^. Variants were called from BAM files using GATK HaplotypeCaller^44^ per chromosome, merged using CatVariants and CombineGVCFs options, then genotyped using GenotypeGVCFs. The dataset was pruned using VCFtools 0.1.16^45^, keeping only biallelic SNPs, and removing indels and outliers in the depth of coverage (only keeping variants within the 10 × −100 × range, with the higher depth variation for low coverage samples). The resulting dataset of 93,538,239 variants served as the basis of all downstream analysis and further data pruning. Approximately 26,228,350 biallelic SNPs were identified across only the bison samples. Further pruning of these datasets was performed using VCFtools^45^ and Plink^46,47^, see below for details. In some analyses, population assignment was given based on the sampling location and whether they were historical (before 1937) or modern (1990-present) samples (Table 1).

### Population genetics and hybridization detection

Estimation of inbreeding coefficient, F, was calculated from the bison specific SNPs using VCFtools^45^ (Supplementary Table 1). A principal component analysis (PCA) was performed to establish patterns in the data between individuals and populations with multidimensional scaling based on 4,393,130 SNPs filtered at a r^2^ value of 0.2. Plink v2.0 option–indep-pairwise 50 10 0.2^47^. Eigenvalues and eigenvectors were calculated on pruned datasets of markers in approximate linkage equilibrium excluding the *B. bubalis* sample using Plink v2.0^47^.

fastSTRUCTURE 1.0^48^ was used to determine population structure and potential shared ancestry between herds. GATK 4.1.2.0^44^ and VCFtools 0.1.16^45^ were used to remove all SNPs that showed no variation among the bison samples. The dataset was further thinned to include only variants with a minor allele frequency between 0.2 and 0.8 among bison samples resulting in 8,836,168 variants. The thinned dataset was then transformed into Plink format^46^. The resulting file was used to test the number of distinct populations K = 2 though K = 12, which were then visualized using Distruct^49^.

TreeMix 1.13^50^ was used to determine the directionality of transfer of haplotypes between populations. All modern bison were assigned to their respective populations while historical samples were included as individuals and a representative of each of the four cattle breeds were assigned to a cattle population. Iterations were run allowing one to eight migration edges on a dataset thinned to one SNP per 1000 base pairs resulting in 1,693,733 SNPs.

Patterson’s D, or D-statistics, were calculated and analyzed by running the ABBA-BABA test using ANGSD^51^. D-statistics are based on all variants across alignment files (BAM) for each sample. Results were ranked by Z scores and the non-significant results were discarded (*p* > 0.05) (Table S2). A negative D-statistic suggests that H3 is closer to H1 than H2, while a positive D-statistic points to H3 being closer to H2 than H1. nABBA values reveal how many blocks of variants suggested H3 was closer to H2, while nBABA values show how many times H3 was closer to H1. Three individuals were selected as visual representatives based on historical representation (HistW_1937), documented population history (Yellowstone_02) or known cattle introgression based on mitochondrial DNA analysis (SantaCataIsl_02). The D-statistics for these three representatives (H1) were compared across all comparisons of bison (H2) and Angus_01 (H3).

HybridCheck was used to identify regions of genomic introgression originating from domestic cattle in bison sequences^24^. A consensus sequence was created using ANGSD doFasta with the option to choose a random base pair at all heterozygous sites^52^. A multiple alignment file per autosome was made from these outputs that included all *B. bison* samples and the *Bos taurus* reference genome and used as input for HybridCheck^24^. The parameters to scan triplets for introgression signal were a window size of 1000 bp, step size of 1 bp. The *Bos taurus* reference and a reference bison from each of the modern populations (Caprock_01, ElkIslandP_01, ElkIslandW_01, Mackenzie_01, SantaCataIsl_02, Vermejo_01, WindCave_02, and Yellowstone_02) were used to compare sequence similarity in triplets for the 24 other *B. bison* used in this study. Introgression blocks were determined as any region where the sequence similarity between the sample and *Bos taurus* reference is greater than the sequence similarity between the sample and comparison bison sample. Any putative introgressed regions were merged within 15,000 bp of a neighboring region meeting the same criteria.

### IntrogressionID—(IID)

We developed IntrogressionID (IID) to identify high-confidence introgression signals in bison resting on three hypotheses: (1) Any alleles introgressed from cattle into bison would be present most commonly as a heterozygous site. (2) To be confident in the validity of the introgression signal, the reference allele from the heterozygous bison must only exist in cattle, and (3) the alternative allele must only exist in bison. (github.com/agaricx/IntrogressionID).

IID works as follows: the bison-cattle filtered SNP VCF was binned into 100 kb windows per-bison as well as cumulatively across the population. The per-window totals for the population were used to calculate the population mean and standard deviation. The population totals were z-score transformed using the population mean and population standard deviation and were passed to a probability density function for a normal continuous random variable to obtain a population wide z-score threshold of significance for every window (Extended Data Fig. 6). With this threshold of significance, the per-window SNP counts for each bison sample were z-score transformed using the population mean and sample standard deviation, and windows were subsequently labeled as either introgressed or not introgressed based on their z-score value (z ≥ 5). Regions were plotted as a whole genome heatmap to visualize significant regions of introgression. Finally, the results from IID were compared to the results from HybridCheck and overlapping regions of introgressed signal were recorded (Supplementary Table 6).

### Data availability

Illumina short read data is deposited in the Sequence Read Archive at the National Library of Medicine under the BioProject accessions PRJNA658430 and PRJNA824118.

## Supplementary Information

Supplementary Information 1.

Supplementary Information 2.
