# Supplementary material for: Genomic evaluation of hybridization in historic and modern North American Bison (Bison bison)
Source: Sci Rep. 2022 Apr 16;12:6397. doi: 10.1038/s41598-022-09828-z (PMC9013353; doi:10.1038/s41598-022-09828-z)
Supplement: Supplementary file 1 — Supplementary Information 1. [file 41598_2022_9828_MOESM1_ESM.pdf]

a

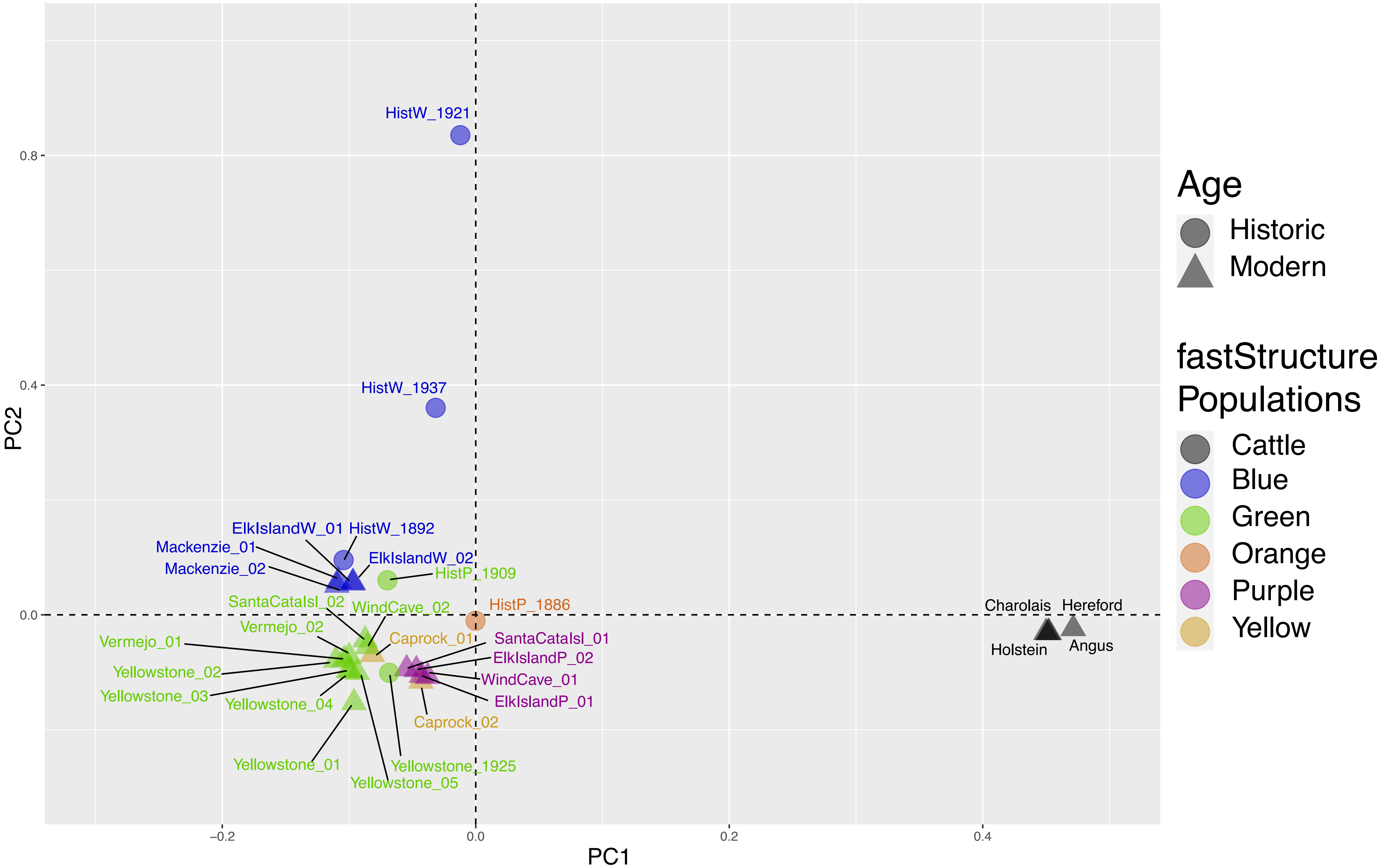


Extended Data Fig. 1 (a) A Principle component analysis (PCA) plot using whole genome SNP data for all bison samples and one representative of the four breeds of cattle used in this study. Samples are color-coded by population according to fastStructure colors. Shapes are used to differentiate historic (before 1940) and modern (after 1990) samples. Based on 4.4 million SNPs.

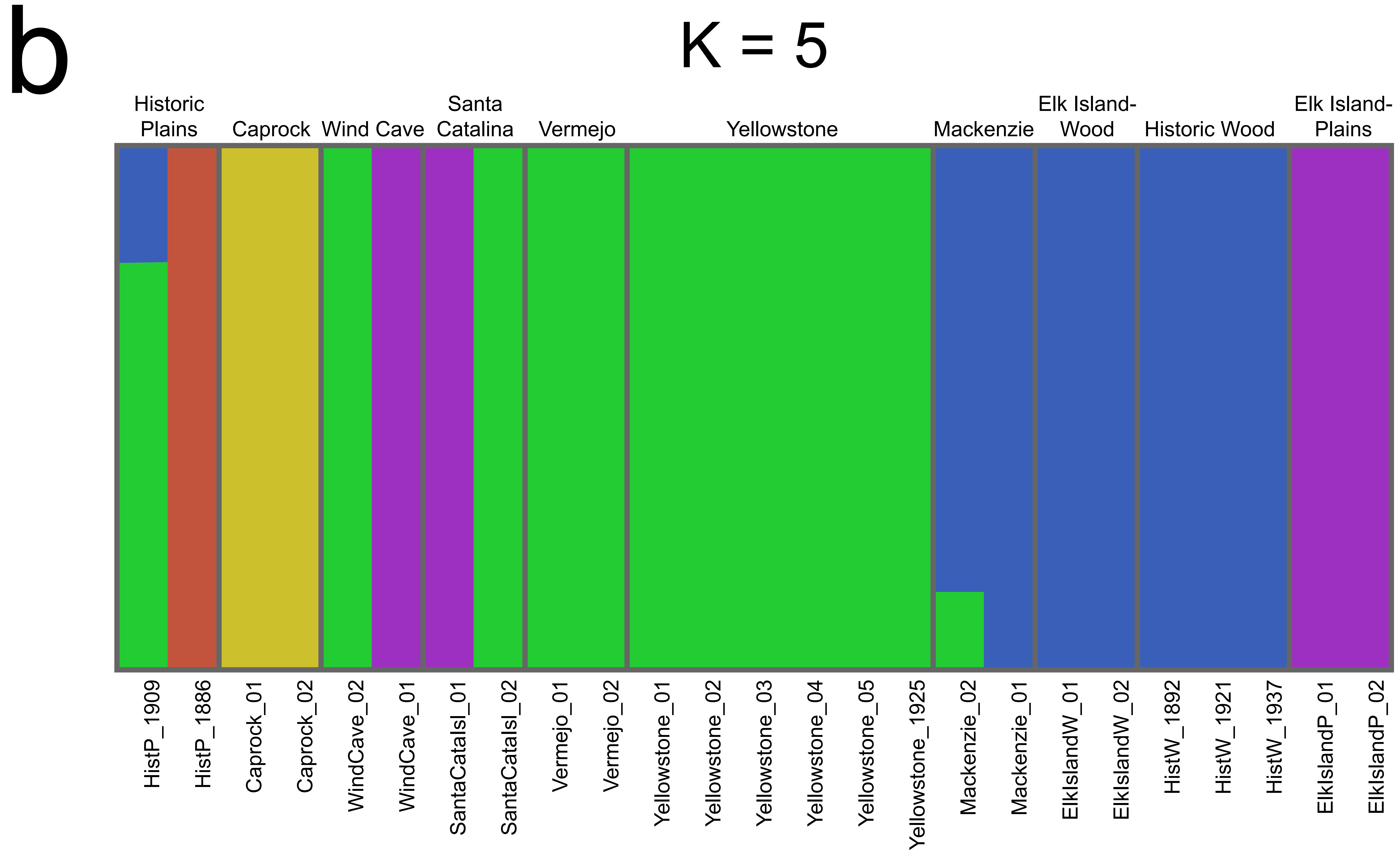


**Extended Data Fig. 1 (b)** fastStructure clustering for K=5, the number of likely distinct population, for all bison based on 8.8 million SNPs.

# fastSTRUCTURE Clustering for K=2 to K=12
# Bison only

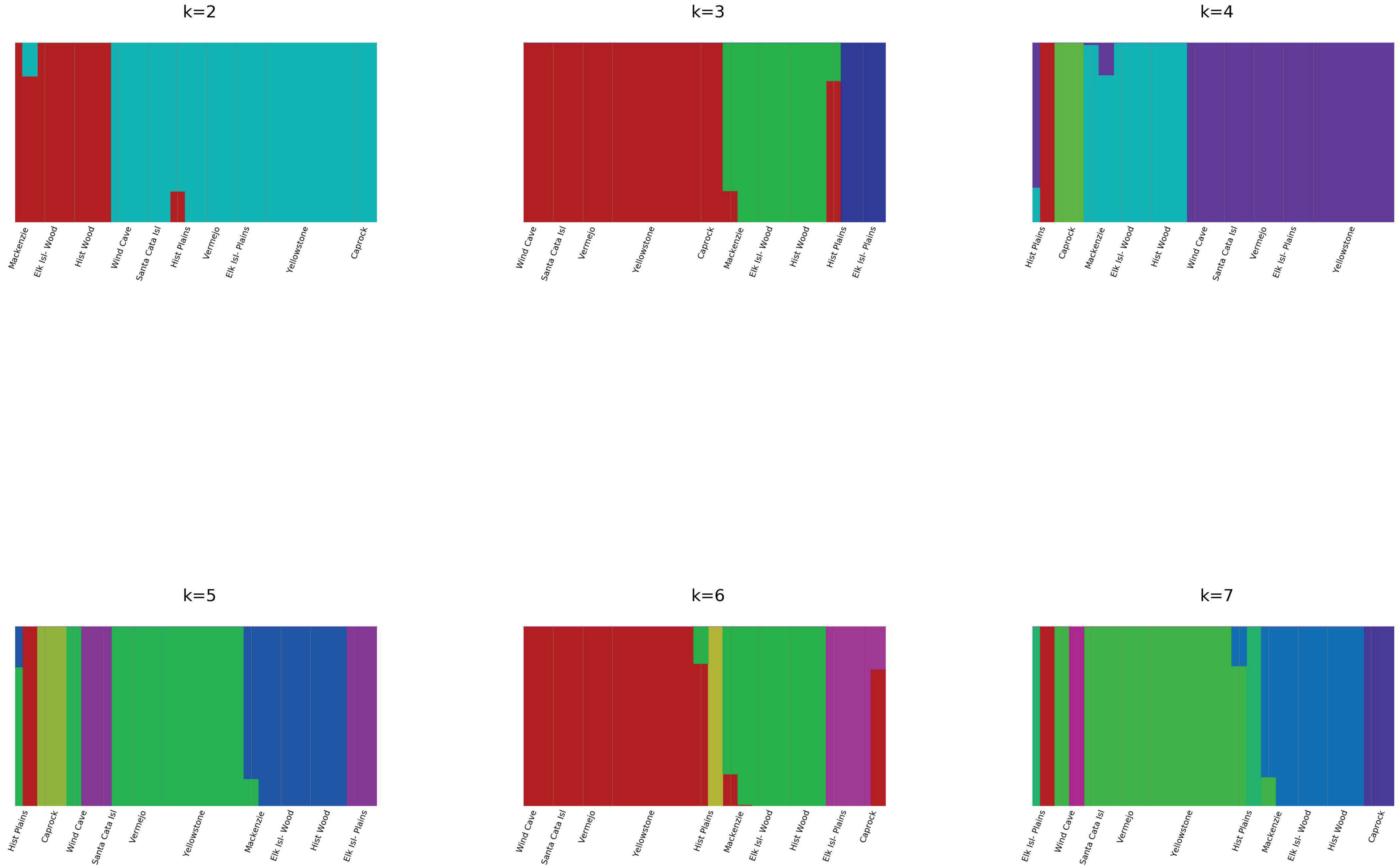


**Extended Data Fig. 2.** fastSTRUCTURE clustering for K=2 through K=12 for all bison based on 8.8 million SNPs.

# fastSTRUCTURE Clustering for K=2 to K=12
# Bison only

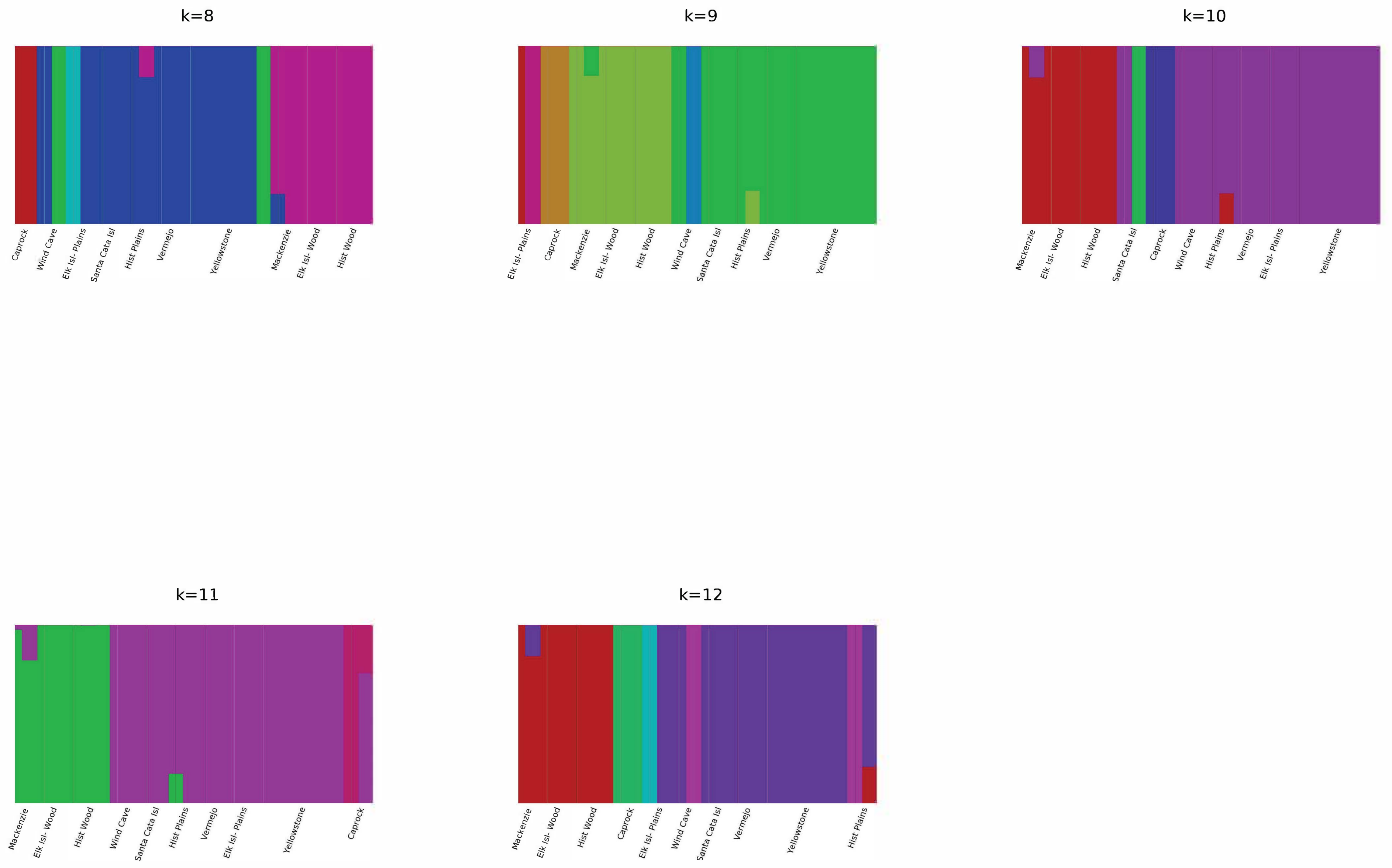


**Extended Data Fig. 2 (continued).** fastSTRUCTURE clustering for K=2 through K=12 for all bison based on 8.8 million SNPs.

# fastSTRUCTURE Clustering for K=2 to K=5 with Cattle Included

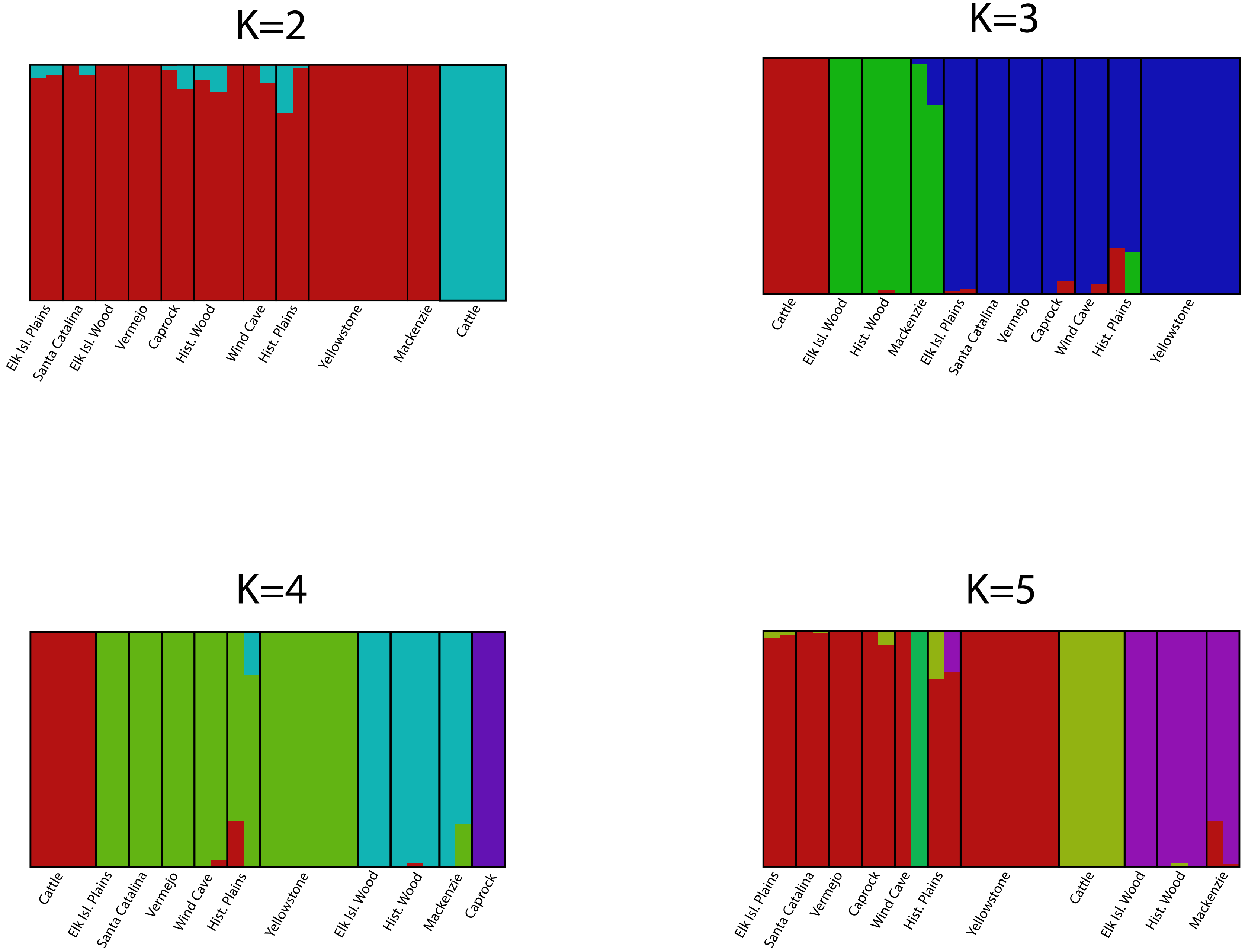


**Extended Data Fig. 2 (Continued).** fastSTRUCTURE clustering for K=2 to K=5 for all bison samples and one cattle per breed (Angus, Charolais, Hereford, and Holstein) based on 8.8 million SNPs.

# Sequencing Depth vs Heterozygous Sites

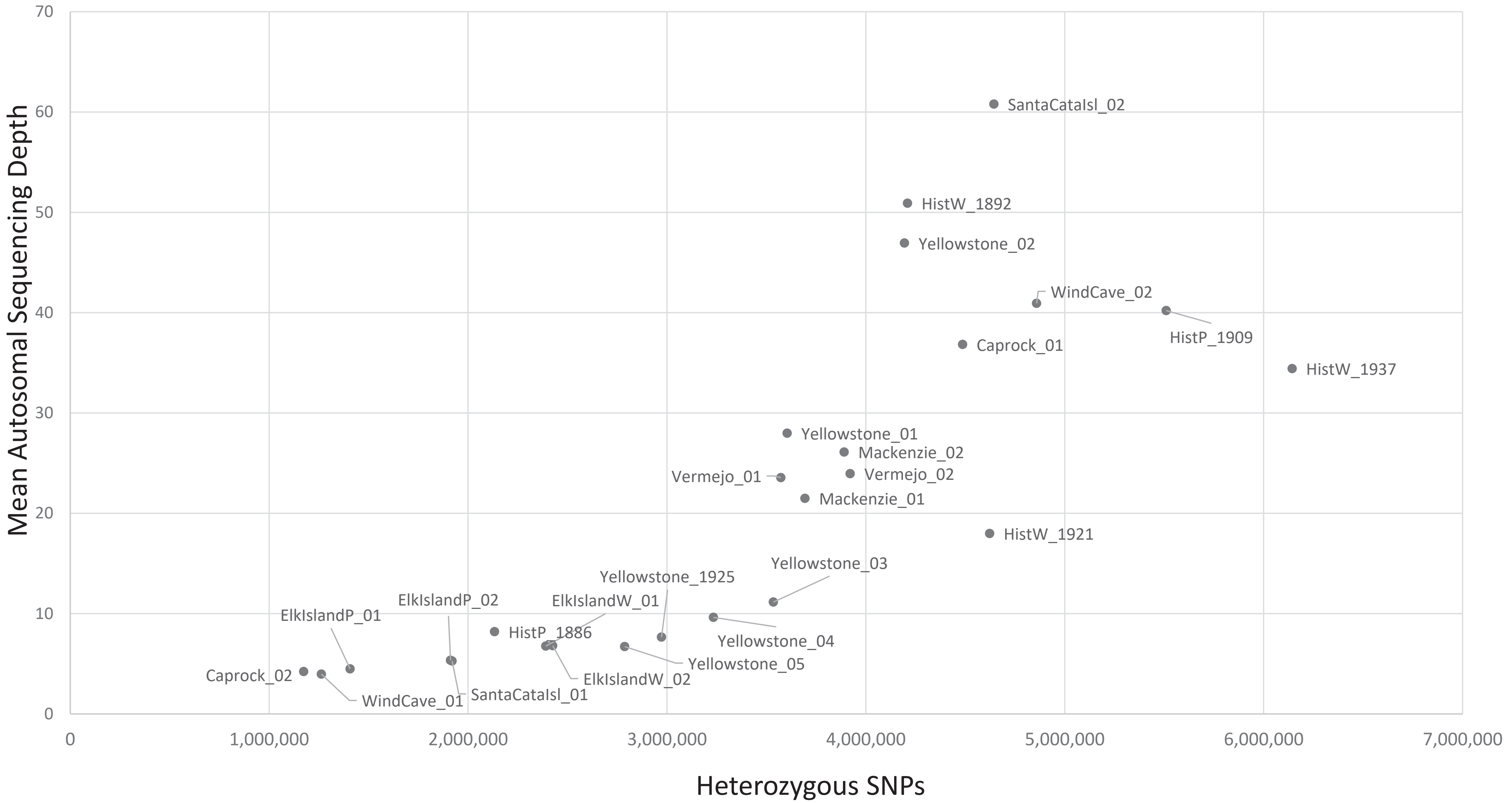


**Extended Data Fig. 3.** The mean autosomal sequencing depth versus the number of heterozygous sites.

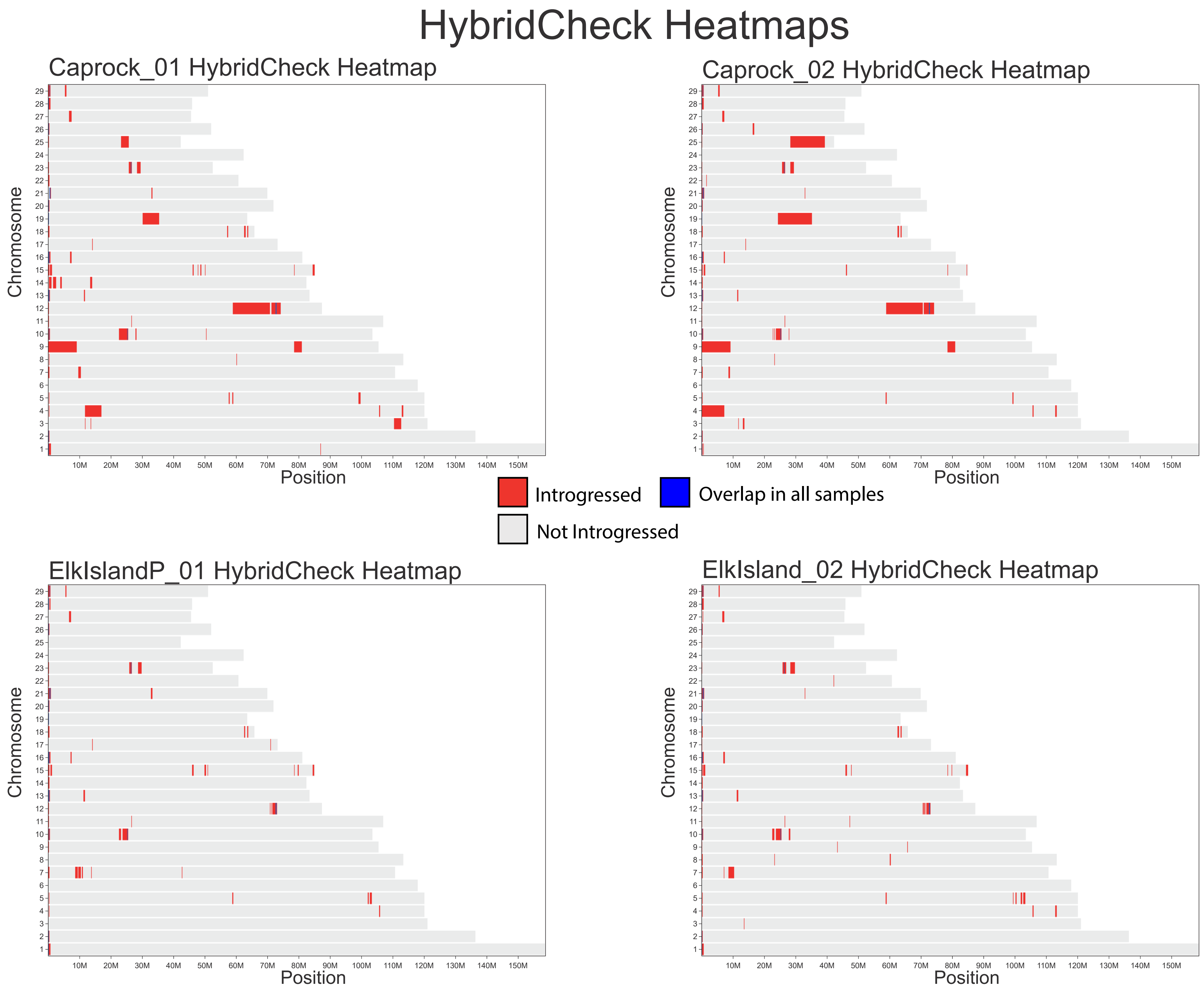


**Extended Data Fig. 4.** Whole genome heatmap of detected introgressed blocks by HybridCheck per sample.

# HybridCheck Heatmaps

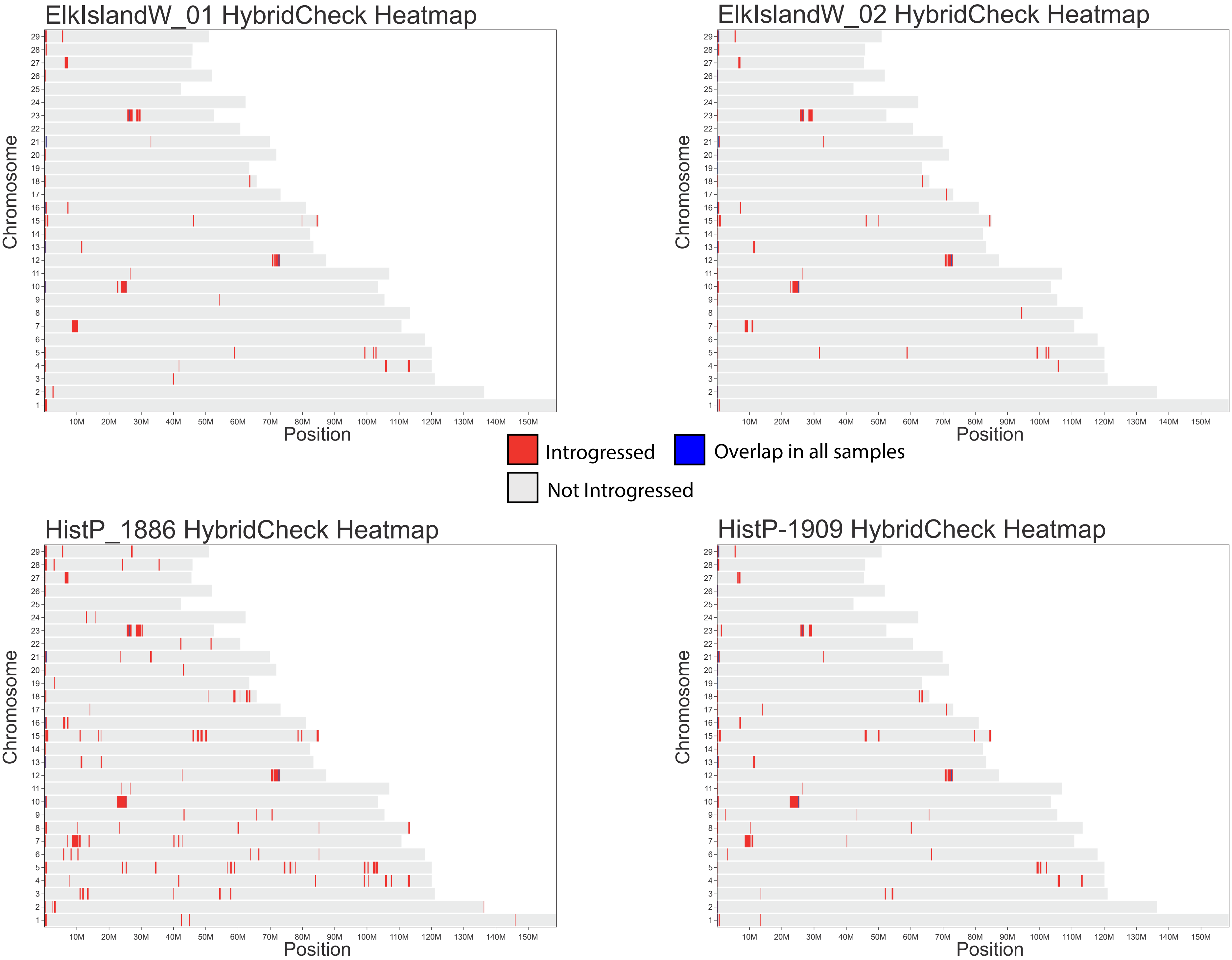


**Extended Data Fig. 4 (continued).** Whole genome heatmap of detected introgressed blocks by HybridCheck per sample.

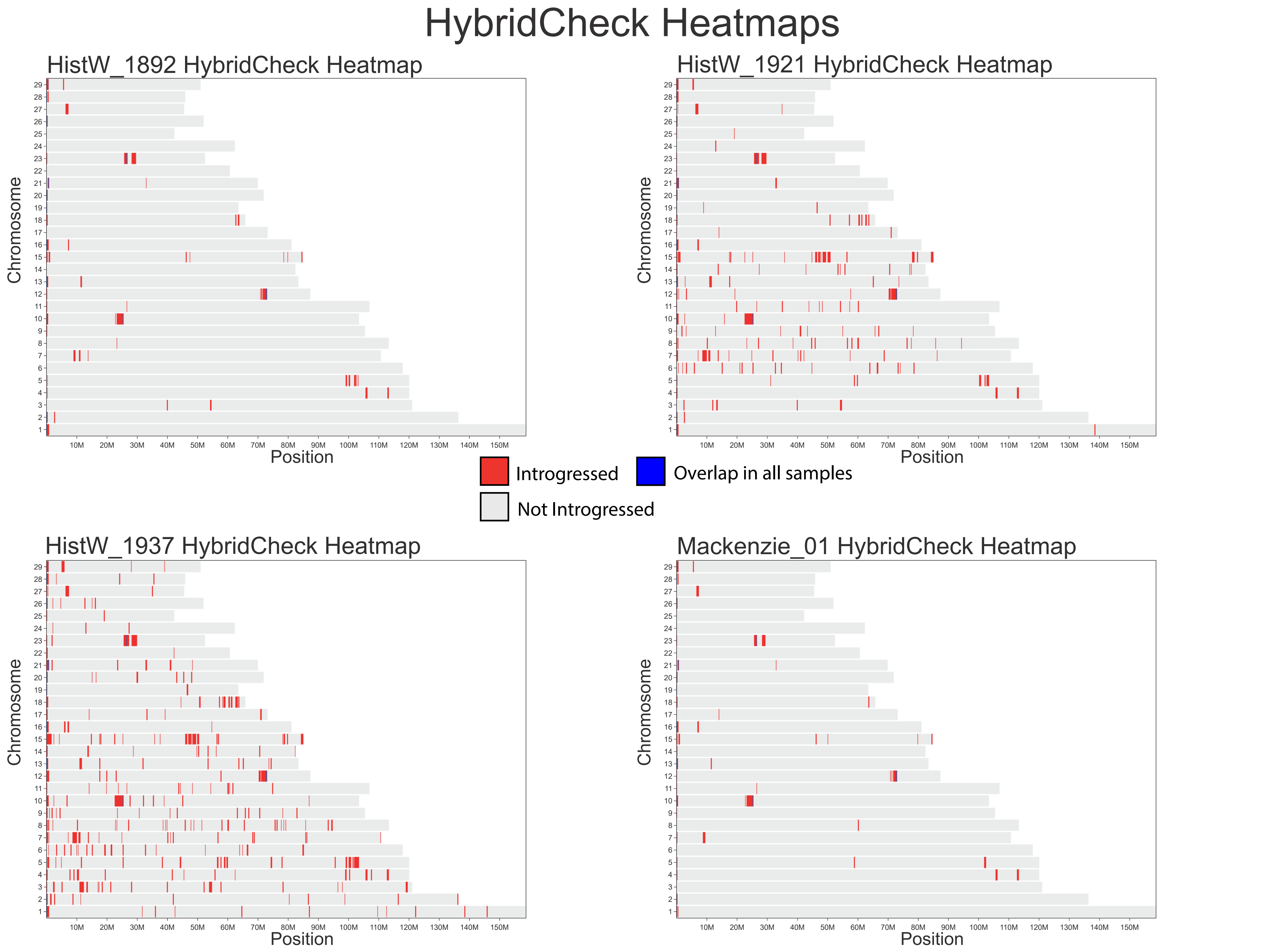


**Extended Data Fig. 4 (continued).** Whole genome heatmap of detected introgressed blocks by HybridCheck per sample.

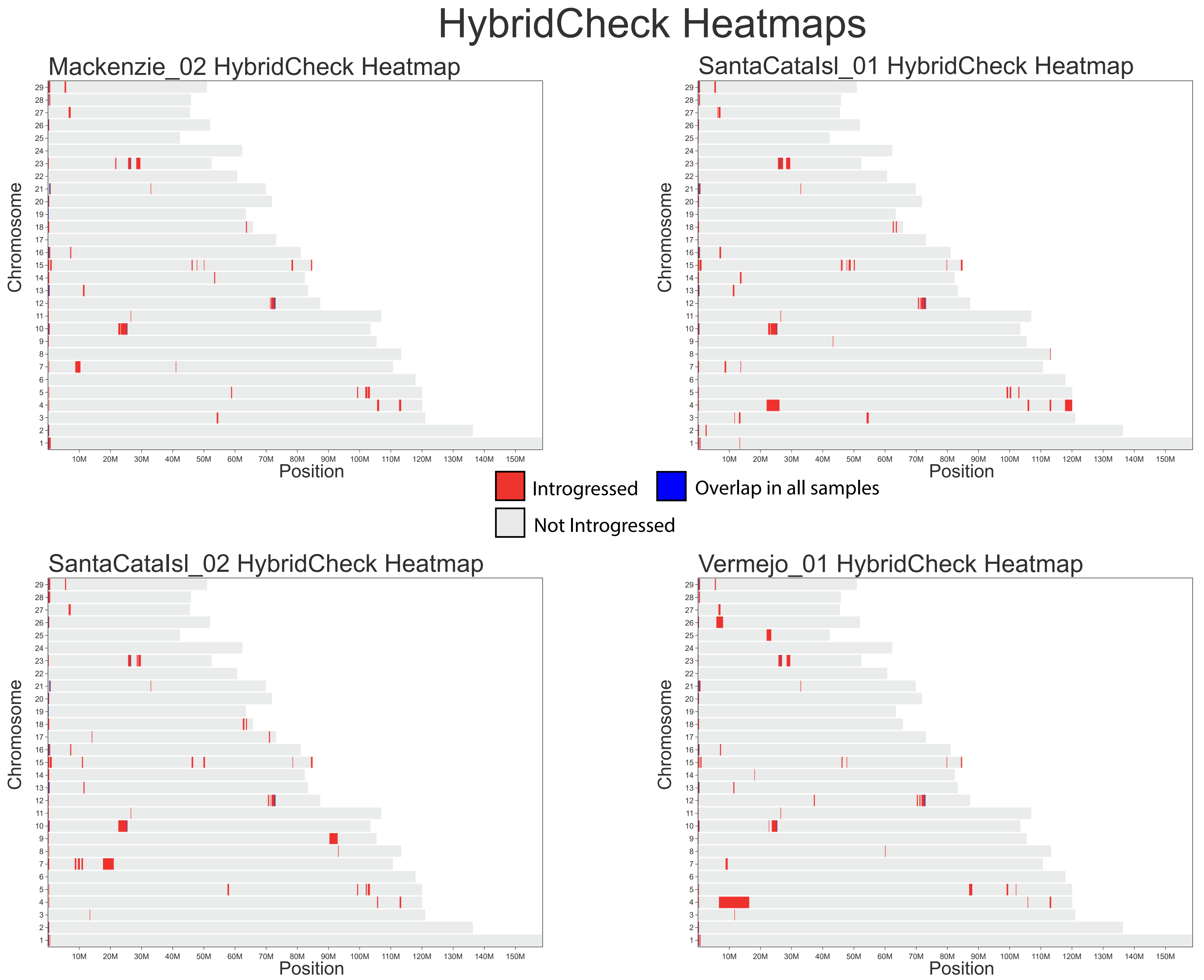


**Extended Data Fig. 4 (continued).** Whole genome heatmap of detected introgressed blocks by HybridCheck per sample.

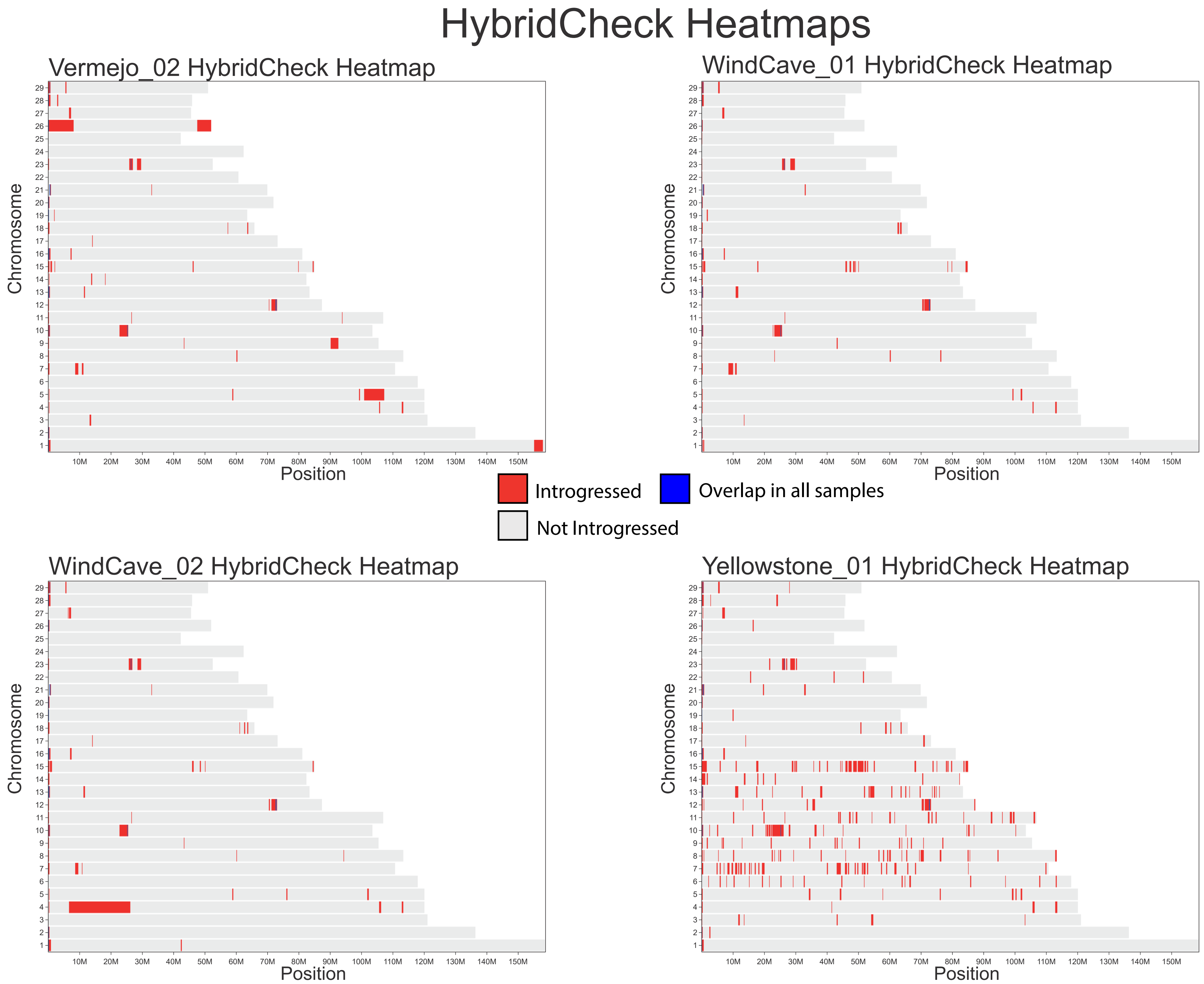


**Extended Data Fig. 4 (continued).** Whole genome heatmap of detected introgressed blocks by HybridCheck per sample.

# HybridCheck Heatmaps

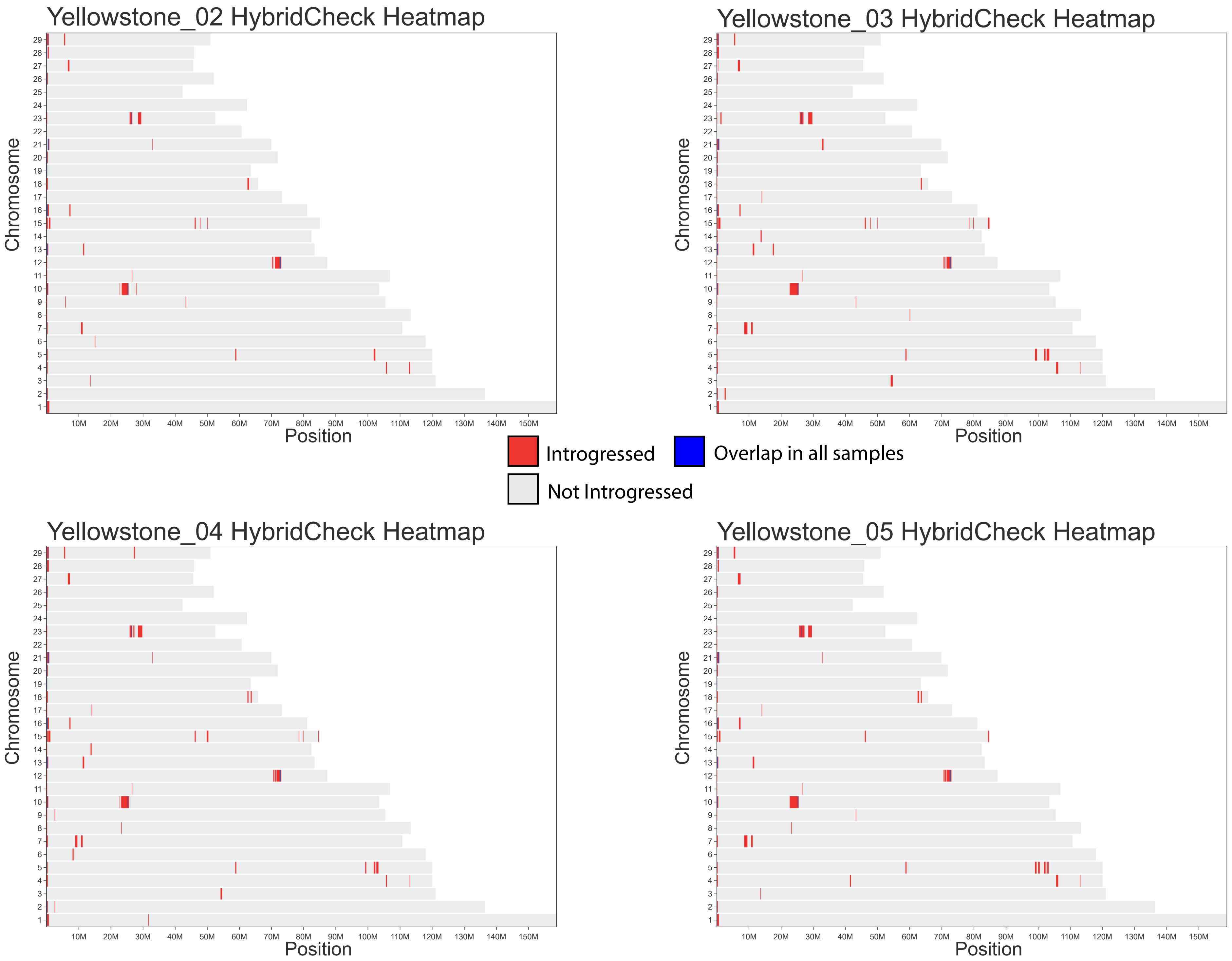


**Extended Data Fig. 4 (continued).** Whole genome heatmap of detected introgressed blocks by HybridCheck per sample.

# HybridCheck Heatmaps

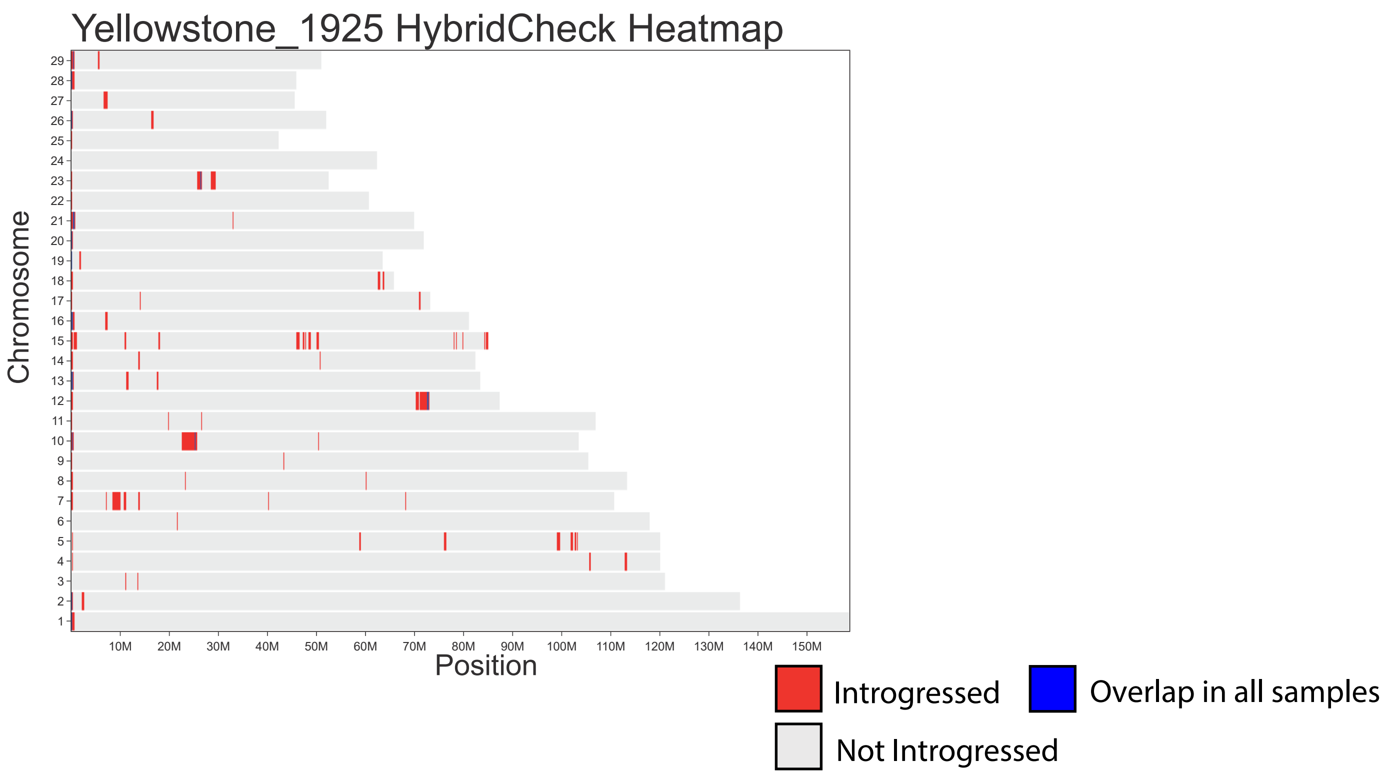


**Extended Data Fig. 4 (continued).** Whole genome heatmap of detected introgressed blocks by HybridCheck per sample.

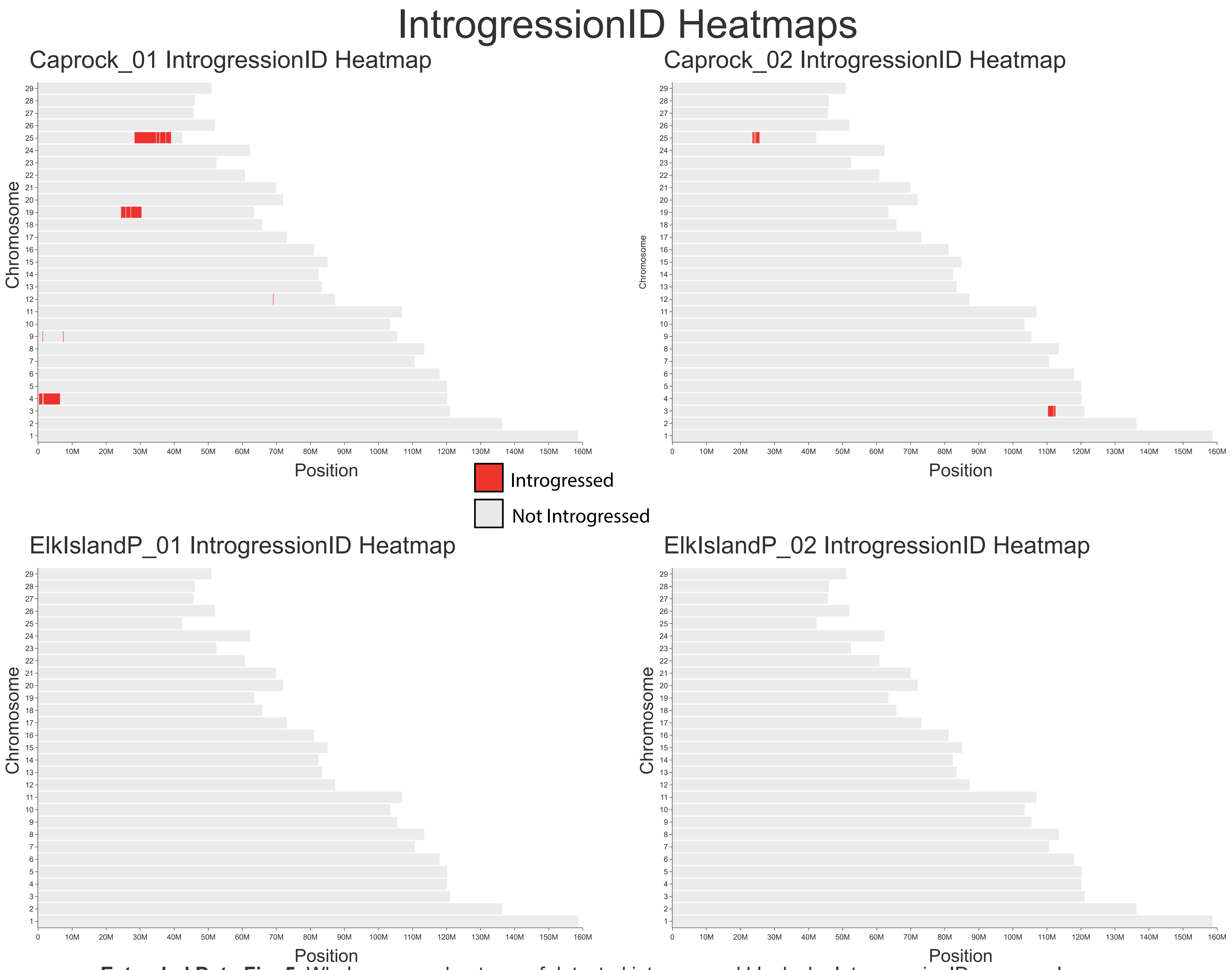


**Extended Data Fig. 5.** Whole genome heatmap of detected introgressed blocks by IntrogressionID per sample.

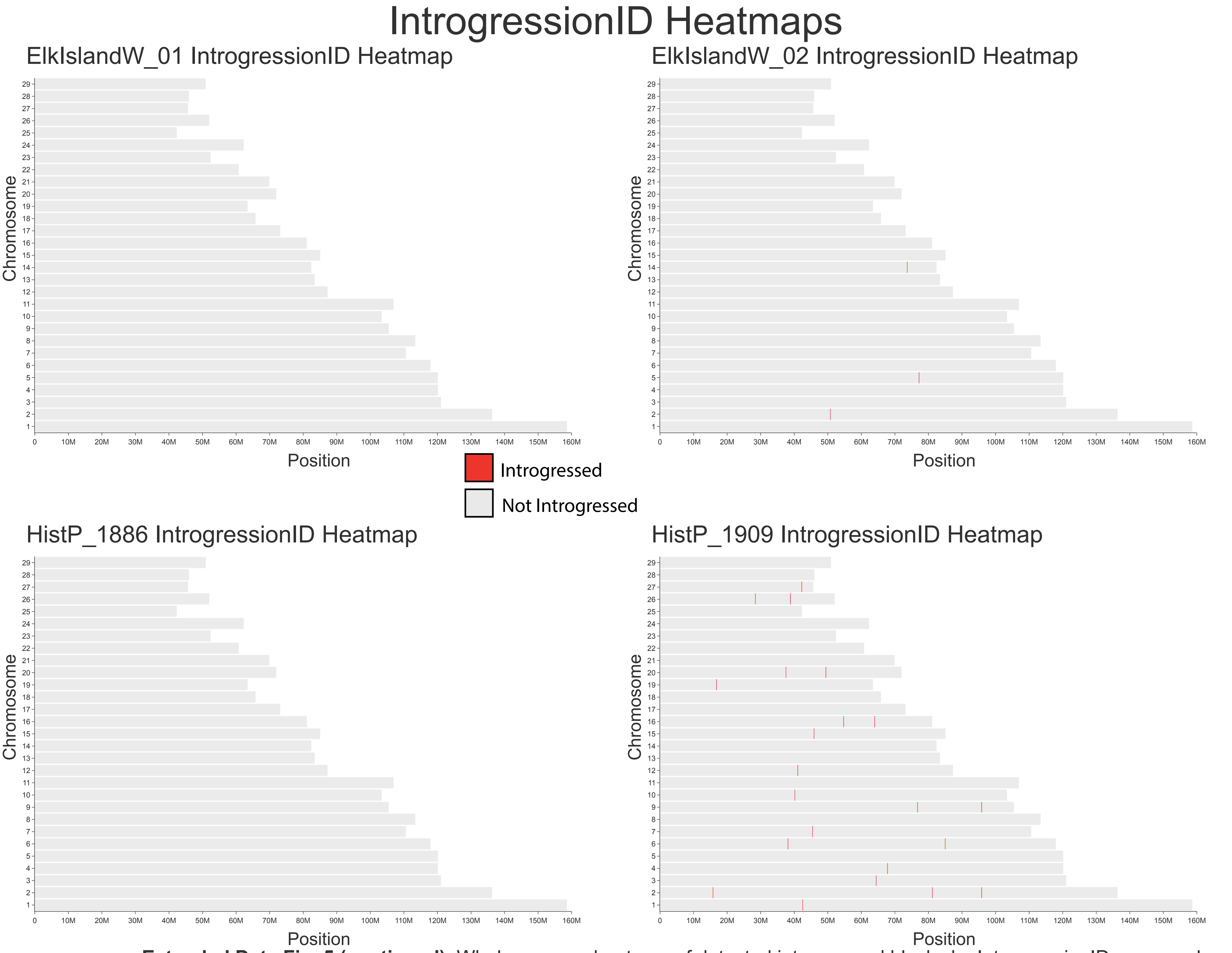


**Extended Data Fig. 5 (continued).** Whole genome heatmap of detected introgressed blocks by IntrogressionID per sample.

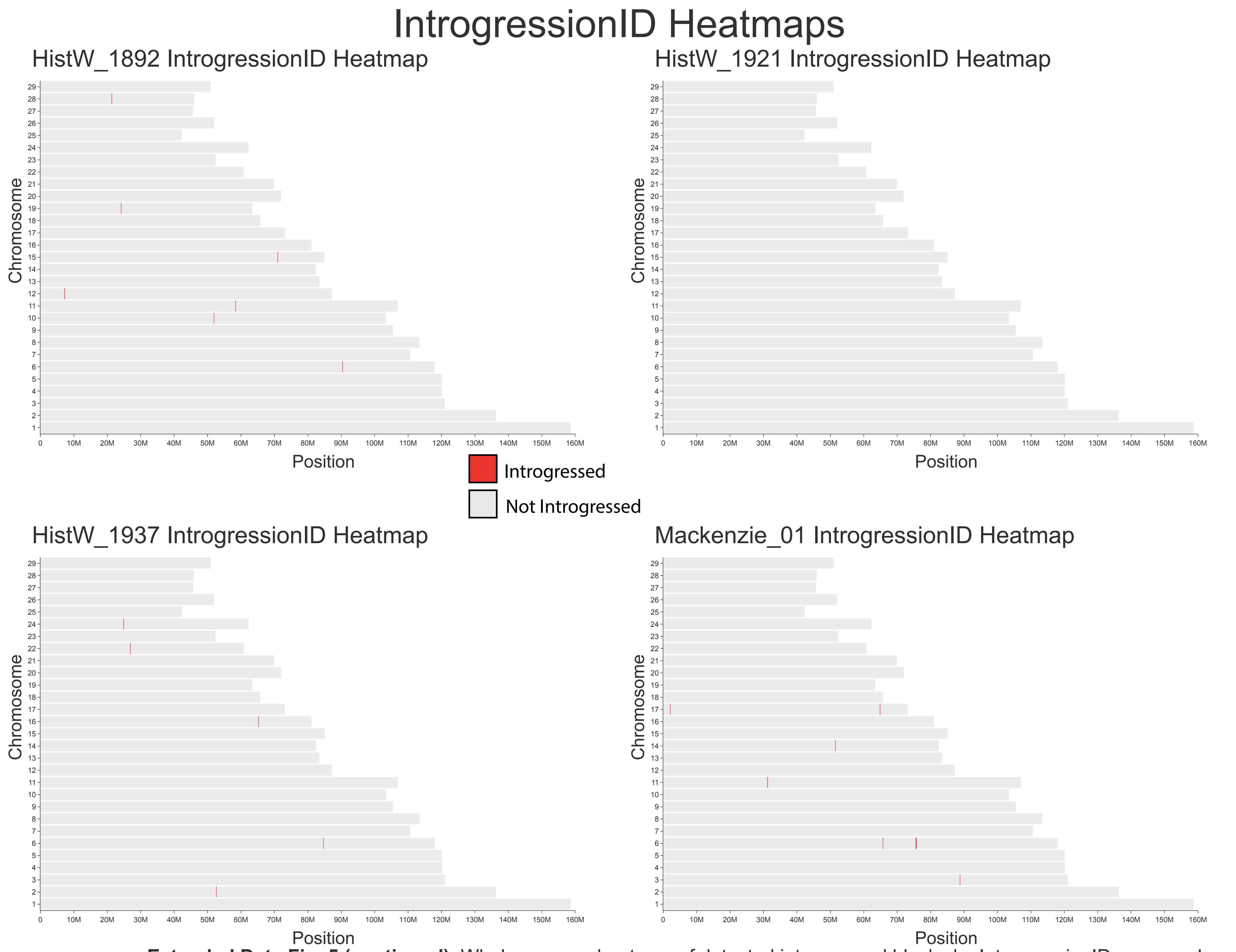


**Extended Data Fig. 5 (continued).** Whole genome heatmap of detected introgressed blocks by IntrogressionID per sample.

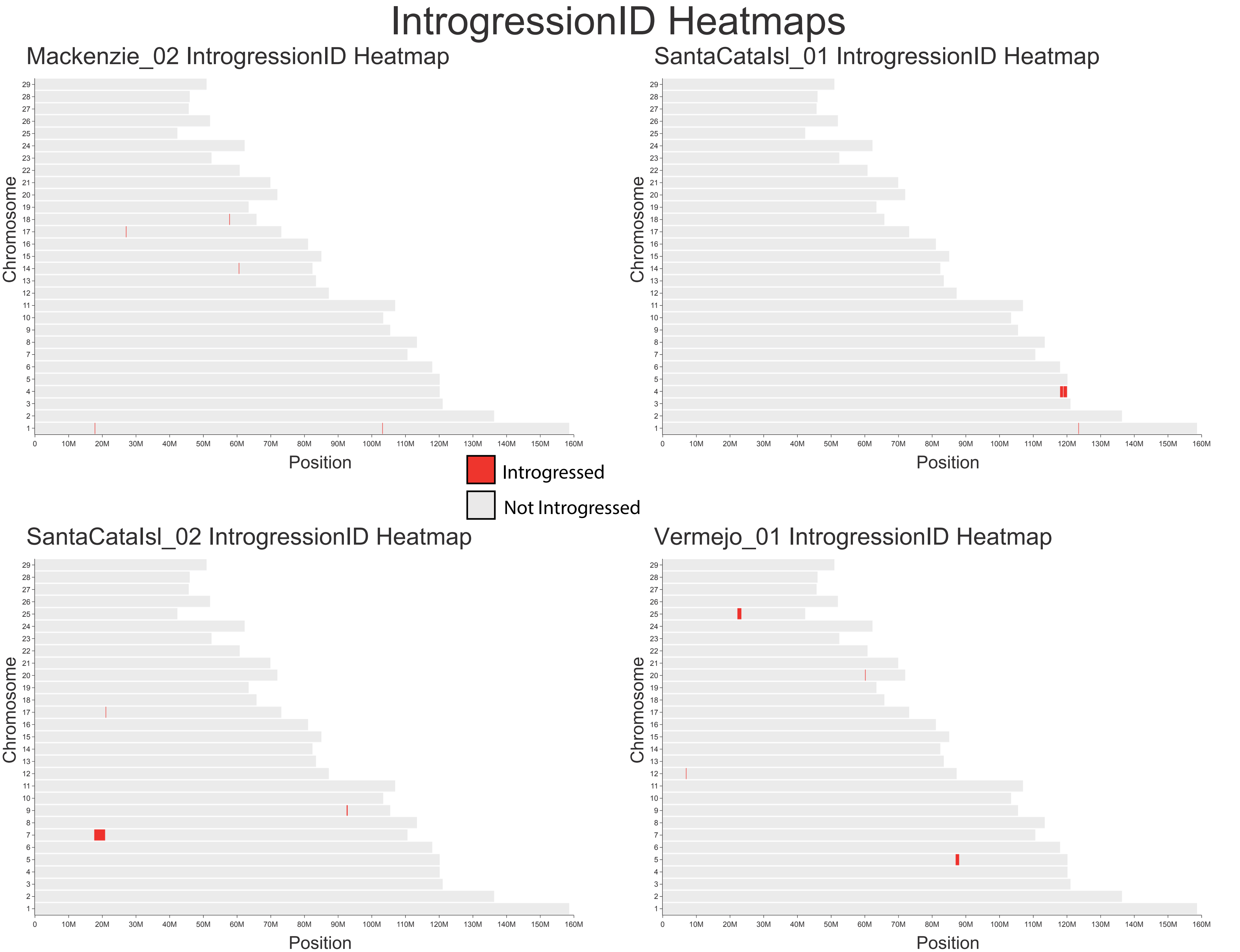


**Extended Data Fig. 5 (continued).** Whole genome heatmap of detected introgressed blocks by IntrogressionID per sample.

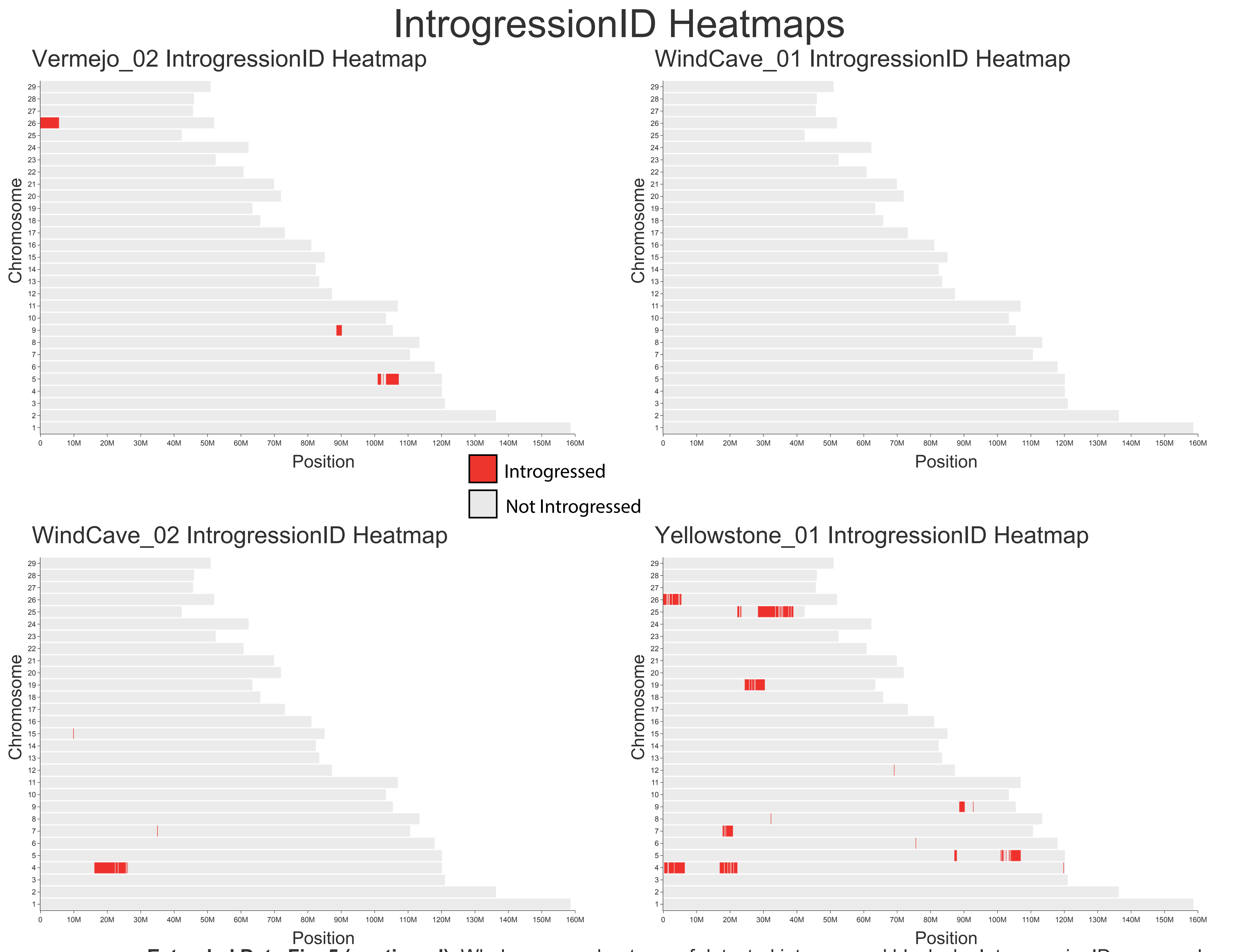


**Extended Data Fig. 5 (continued).** Whole genome heatmap of detected introgressed blocks by IntrogressionID per sample.

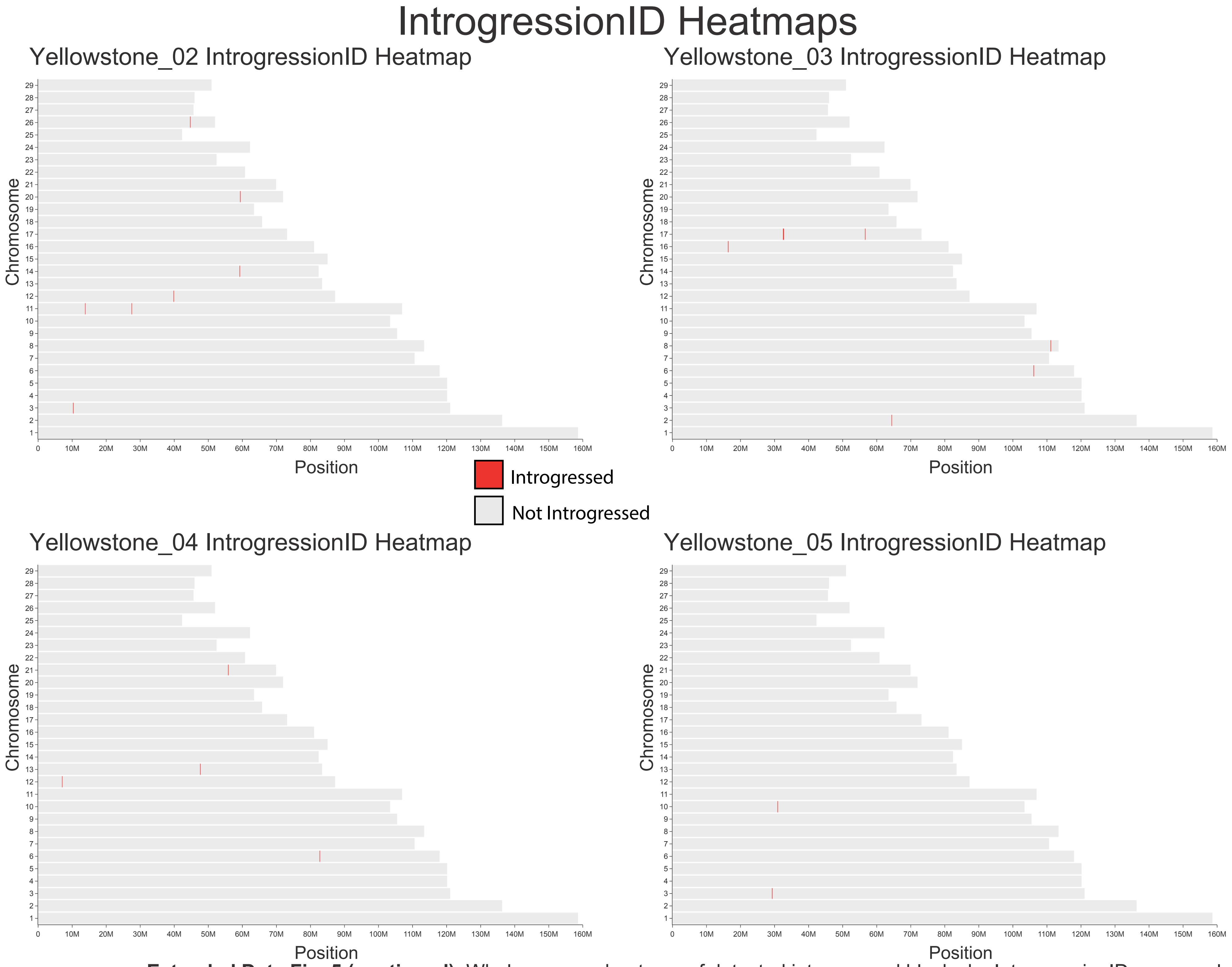


**Extended Data Fig. 5 (continued).** Whole genome heatmap of detected introgressed blocks by IntrogressionID per sample.

# IntrogressionID Heatmaps

## Yellowstone_1925 IntrogressionID Heatmap

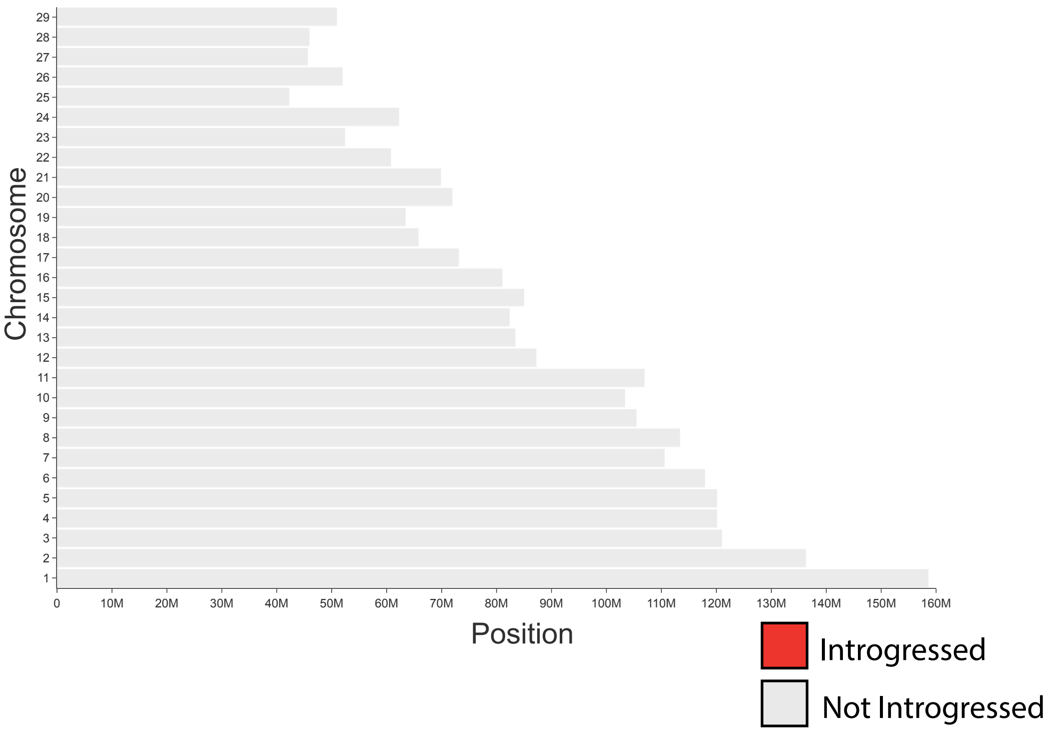


**Extended Data Fig. 5 (continued).** Whole genome heatmap of detected introgressed blocks by IntrogressionID per sample.

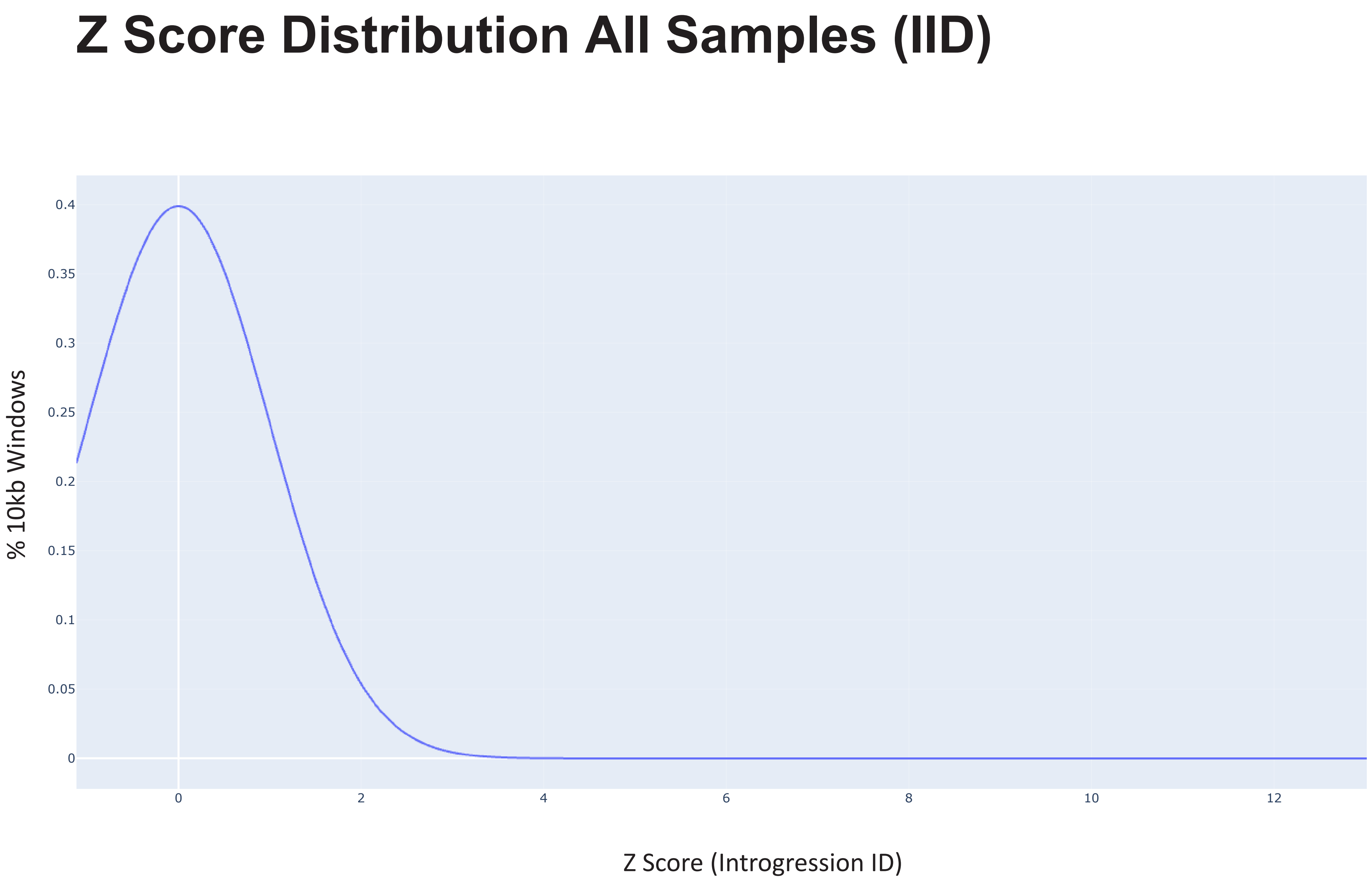


**Extended Data Fig. 6.** Probability density plot of population wide per-window z-scores. The plot was used to determine a population wide threshold of significance to be used to identify windows of significant introgression signal in each bison sample.
